# Ocean emission of microplastic

**DOI:** 10.1093/pnasnexus/pgad296

**Published:** 2023-10-03

**Authors:** Daniel B Shaw, Qi Li, Janine K Nunes, Luc Deike

**Affiliations:** Department of Mechanical and Aerospace Engineering, Princeton University, Princeton, NJ 08544, USA; School of Civil and Environmental Engineering, Cornell University, Ithaca, NY 14853, USA; Department of Mechanical and Aerospace Engineering, Princeton University, Princeton, NJ 08544, USA; Department of Chemical and Biological Engineering, Princeton University, Princeton, NJ 08544, USA; Department of Mechanical and Aerospace Engineering, Princeton University, Princeton, NJ 08544, USA; High Meadows Environmental Institute, Princeton University, Princeton, NJ 08544, USA

**Keywords:** microplastics transport, bubble bursting, ocean emissions, air-sea interactions

## Abstract

Microplastics are globally ubiquitous in marine environments, and their concentration is expected to continue rising at significant rates as a result of human activity. They present a major ecological problem with well-documented environmental harm. Sea spray from bubble bursting can transport salt and biological material from the ocean into the atmosphere, and there is a need to quantify the amount of microplastic that can be emitted from the ocean by this mechanism. We present a mechanistic study of bursting bubbles transporting microplastics. We demonstrate and quantify that jet drops are efficient at emitting microplastics up to 280μm in diameter and are thus expected to dominate the emitted mass of microplastic. The results are integrated to provide a global microplastic emission model which depends on bubble scavenging and bursting physics; local wind and sea state; and oceanic microplastic concentration. We test multiple possible microplastic concentration maps to find annual emissions ranging from 0.02 to 7.4—with a best guess of 0.1—mega metric tons per year and demonstrate that while we significantly reduce the uncertainty associated with the bursting physics, the limited knowledge and measurements on the mass concentration and size distribution of microplastic at the ocean surface leaves large uncertainties on the amount of microplastic ejected.

**Significance Statement**

Microplastic particles are increasingly prevalent in the ocean. Bursting bubbles are known to transmit salt crystals and organic materials from the ocean into the atmosphere: influencing the radiative balance and serving as cloud condensation nuclei. We demonstrate through laboratory experiments that bursting bubble jet drops can scavenge and eject microplastic (10 to 280μm in size) to the air. Results are then integrated globally, accounting for how air bubbles are produced and ocean measurements of microplastic, to estimate the global ocean microplastic emission which ranges from 0.02 to 7.4 Mt/yr with a best guess of 0.1 Mt/yr. We show that the remaining uncertainty resides in the limited knowledge of ocean microplastic.

## Introduction

The increasing prevalence of plastics in the ocean is a global-scale issue with wide-ranging impacts. Considerable scientific focus has been placed on ocean plastic debris since the 1970s (1) with an estimated 10% of all plastic produced eventually being deposited into the ocean (2). Microplastic, typically defined as plastic particles of size between 1μm and 5 mm (3), is found in the atmosphere, and recent papers suggest that ocean is a potentially significant source of atmospheric microplastic (4–6). Estimates range from 0 to 22 mega metric tons, Mt, per year (5, 7); at the upper range, oceans would be one of the largest sources of atmospheric microplastic (5), while other studies estimate that microplastic emissions by the ocean are negligible (7). As such, understanding microplastic emission by the ocean is an urgent unmet need to close global plastic budgets (3, 5).

Plastic debris can be found in all marine environments (8) and are being transported by ocean currents (9, 10) and waves (11). It is estimated that 19 to 23 Mt of plastic is currently flowing into the ocean annually (12, 13). If current trends continue, it is expected that by 2040 the annual rate of microplastic entering aquatic environments from land will have increased by 260% from 2016 (14) and a “peak plastic waste” is not expected to be reached until 2100 (15). Their increasing pervasiveness in the world’s environments presents a serious issue motivating the accurate quantification of the microplastics cycling in and out of the ocean.

Material such as water, salts, and biological material have long been known to be transported from the ocean to the atmosphere via sea spray droplets (16–20). The rate of transport is high enough to affect global climate dynamics; salt crystals and organic aerosols influence the radiative balance of the atmosphere and serve as cloud condensation nuclei (21–24).

Sea spray aerosols are generated by two pathways: spume drops resulting from high wind shearing of wave crests (19, 25) and surface bubbles bursting (16, 26)—itself decomposed into film and jet drops (16, 17). Film drops come from the liquid that was in the bubble’s thin-film cap at the moment of bursting. Their size is controlled by the bubble’s radius and the cap thickness at burst, (17, 27) and they are responsible for most submicron spray drops (16, 19, 28). Jet drops are formed from the collapse of the bubble’s underwater cavity and are responsible for most super-micron drops (16, 19, 29); capillary waves travel down the bubble’s empty cavity to focus and form a jet which destabilizes into droplets (30–34).

As bubbles rise to the surface, they scavenge soluble and insoluble material (18, 35–40), leading to an enhancement of the material’s concentration in the ejected droplet. The efficiency factor *E*, by which the concentration in the drop changes, increases with the height a bubble rises to the surface, *H*; a linear scaling is predicted by a simple interceptor model (41, 42). However, laboratory experiments have reported a decrease in the rate of material collection with increasing *H* (18, 43, 44), which can be related to saturation of the bubble’s surface area with particles (35) and decreasing surface mobility of the bubble as it rises due to the scavenging of surface-active molecules (43). Correction factors have been proposed for the scavenging of nonspherical bacteria (35), and the origin of liquid in the jet drop has been modeled numerically (36).

**Fig. 1.**
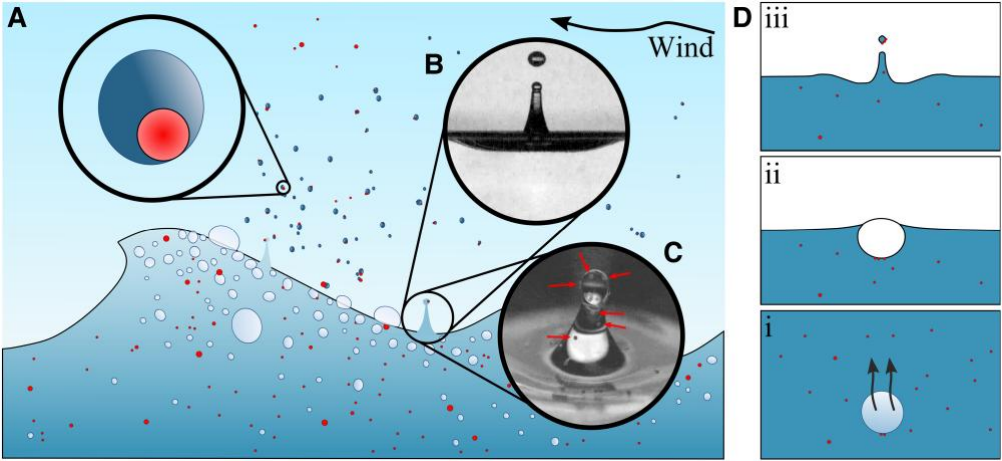
Sketch of the relevant processes ejecting microplastic out of the ocean adapted from Refs. (4, 16). A) Microplastic (red/darker color) in the ocean is transported into the atmosphere by sea spray drops. B) Bursting bubbles create small drops or aerosols such as jet drops. C) Microplastics present in the liquid can be carried up into the jet drops produced. The arrows point to 100μm microplastic pieces. Drops produced can be picked up by wind and carry microplastic material up into the atmosphere. The liquid eventually evaporates leaving behind microplastic pieces. D) The relevant physical processes for bubble-bursting ejection of microplastic start with the scavenging of particles as the bubble rises Di). After arriving at the surface Dii), the bubble eventually settles into its equilibrium shape which—upon bursting—focuses capillary waves at its base to form jet drops Diii) which carry microplastic material.

In this work, we characterize and quantify how microplastic is ejected from the ocean by bubble bursting as shown in Fig. 1. An experimental study of microplastic ejection by individual bubbles is first presented. By varying the liquid properties, bubble size, depth of bubble rise, microplastic size and concentration, equations for jet drop capture of microplastic are developed. Subsequently, we integrate our findings on the individual transport mechanism into a global estimation of microplastic emissions, by considering a physics-based sea spray generation function for jet drops which is a function of wind and waves at the ocean surface together with estimates of the ocean microplastic concentration (8). Finally, an estimate of the global emission of microplastic from the ocean for multiple possible microplastic concentrations is discussed. We argue that remaining uncertainties reside predominantly in the limited knowledge of ocean microplastic concentration maps.

## Single bubble microplastic ejection

**Fig. 2.**
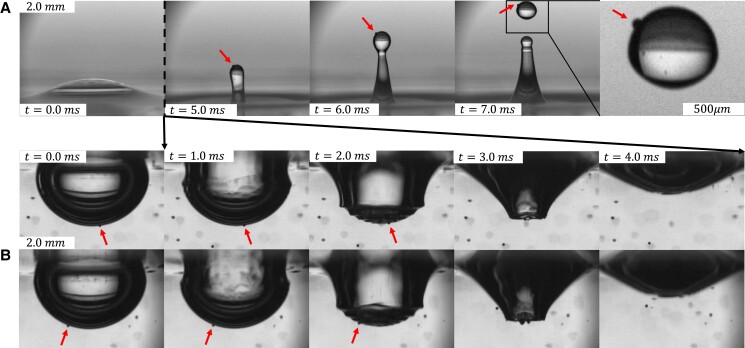
An $R_{b}=1.9$ mm bubble bursting in deionized water ($R_{b}/l_{c}=0.70$ and $R_{b}/l_{\mu }=150,000$) to produce a jet drop, $r_{d}=380\;\mu m$, which can transport 100μm diameter polyethylene ($\rho_{\mathrm{MP}}=1.00\;g/{\mathrm{cm}}^{3}$) microplastic pieces. There are $\chi =3.38\times 10^{8}\;m^{-3}$ pieces of plastic per unit volume of liquid in the bulk surrounding the bubble. In this regime, microplastic pieces are able to be captured and transported by the jet drop, as shown in sequences A and B.

We observe the transport of microplastics by individual bubble bursting through laboratory experiments using high-speed photography as shown in Fig. 2: the top row is an above-surface view, while the two lower sequences (A and B) show an underwater view of two different bursting events at the same conditions (liquid, bubble size, particle size, and particle number concentration). The red arrows in each sequence indicate microplastic particles of interest. The above-water sequence shows the formation of a drop from the jet which carries a clearly visible microplastic particle. In sequences A and B, the highlighted particles are captured even though the particle in B is not at the bottom center of the cavity. Throughout the entire study, no microplastic particles are observed in the thin-film cap which is *O* (0.1−10μm) thick and smaller than the size of particles studied.

### Size of jet drops carrying microplastic

**Fig. 3.**
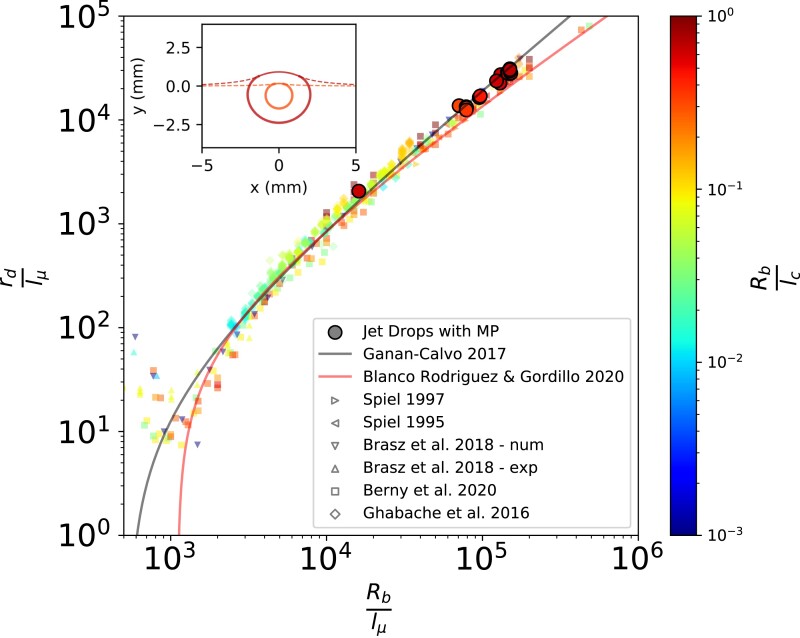
Jet drop size, $r_{d}$, as a function of bubble size, $R_{b}$, both nondimensionalized by the visco-capillary length scale $l_{\mu }=\mu_{l}^{2}/(\rho_{l}\sigma )$. The results from our experiments where microplastic is transported by jet drops (large solid circles) are compared to multiple experimental and numerical studies measuring the size of jet drops (30, 34, 46–50). The shape of a surface bubble changes with size, $R_{b}/l_{c}$, as shown by the inset. The shape of the largest and smallest bubbles in this study are shown in red (darker color) and orange (lighter color), respectively.

The radius, $r_{d}$, (and velocity) of the first jet drop produced by a bursting bubble has been shown to be controlled by the ratio of the bubble radius, $R_{b}$, to the visco-capillary length scale, $l_{\mu }=\mu_{l}^{2}/(\rho_{l}\sigma )$ (45, 46), where $\mu_{l}$ is the liquid viscosity, $\rho_{l}$ is the liquid density, and σ is the surface tension. Measurements of the jet drop radius as a function of bubble size from multiple prior (experimental and numerical) studies (30, 34, 46–50) as well as proposed relationships (46, 47, 51) (see formulas in online supplementary material) are shown in Fig. 3. The radii of jet drops carrying microplastic in the present study are shown in Fig. 3 as solid circles. They agree well with existing data which indicates that the established relations for $r_{d}$ can be applied to the microplastic transport process.

The ratio of the bubble size to the capillary length, $l_{c}=\sqrt{\sigma /(\Delta \rho g)}$, (where Δρ is the density difference between the two fluids and *g* is gravity) determines the bubble shape. The range of bubble sizes in this study is shown in the inset of Fig. 3, and size is a secondary parameter controlling jet drop existence and the speed at which they are ejected (50) (see online supplementary material). The inset of Fig. 3 shows the outline of the largest and smallest bubbles in this study: $R_{b}/l_{c}=0.70$ (red) and $R_{b}/l_{c}=0.31$ (orange).

### Bursting at the surface

Having determined the jet drop radius, we measure the number of microplastic particles captured by each bursting bubble. This is obtained by catching each individual drop on a flat plate suspended above the free surface. The drops are then dried and any particles left behind are counted in a microscope. We measure about 50–100 bubble-bursting events at each condition to account for statistical variability in the system (see Tables S3 and S4 in the online supplementary material).

Microplastic ejection is first studied by releasing bubbles at a fixed height, H=1.5 cm, below the free surface. At this fixed value of small *H* (comparable to the bubble size), the number of particles ejected, *N*, is a function of the particle size, $r_{\mathrm{MP}}$, the particle concentration in the liquid, χ, and the drop size, $r_{d}$, which is determined by $R_{b}/l_{\mu }$ (with $l_{\mu }$ being the visco-capillary length scale accounting for the properties of the liquid, i.e. viscosity, surface tension, and density following Fig. 3). The Stokes number defined as St $=\;(\rho_{\mathrm{MP}}-\rho_{l})r_{\mathrm{MP}}^{2}u_{b}/(9\mu_{l}R_{b})$ with $\rho_{\mathrm{MP}}$ being the particle density and $u_{b}$ the bubble’s rise velocity, describes the particle’s response time to drag forces versus the characteristic timescale of the flow. For all conditions, St≪1 (in fact, for some conditions $\rho_{\mathrm{MP}}=\rho_{l}=1.00\;g/{\mathrm{cm}}^{3}$ such that St≈0). As such, two dimensionless groups are expected to control the number of microplastic particles ejected:


(1) $$N=g(r_{\mathrm{MP}}/r_{d},\chi r_{d}^{3}).$$

**Fig. 4.**
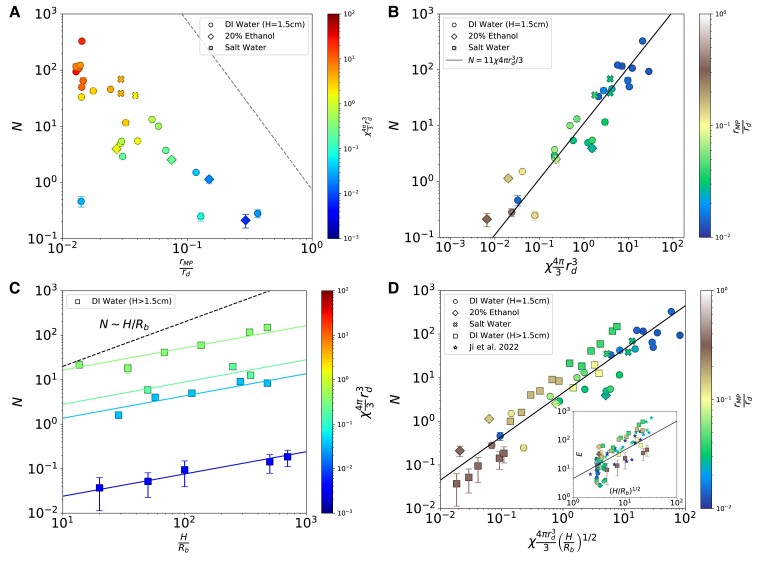
Number of microplastic particles transported by a jet drop, *N*. Data for deionized water, salt water (at ocean salinity), and 20% ethanol are plotted as circles, crosses, and diamonds, respectively, and the error bars are the standard error of *N*. A) Data at H=1.5 cm for *N* versus $r_{\mathrm{MP}}/r_{d}$ color coded by $\chi \frac{4}{3}\pi r_{d}^{3}$, the bulk liquid’s number concentration times drop volume. The gray dashed line represents a theoretical maximum number of particles in the drop. B) Same data with *N* as a function of $\chi \frac{4}{3}\pi r_{d}^{3}$ with $r_{\mathrm{MP}}/r_{d}$ color coded. The solid black line is Eq. 2 with a prefactor of 11 found by best fit. At a constant rise height, the number of microplastics per bubble is well described by the particle concentration and jet drop size. C) *N* as a function of $\left(H/R_{b}\right)$. Grouping the data by consistent color (which denotes $\chi 4\pi r_{d}^{3}/3$ of each point) shows an $N\propto (H/R_{b}{)}^{1/2}$ relationship. The dashed line shows the linear scaling from the interceptor model (41) and does not describe the data with large $H/R_{b}$ values, suggesting that as a bubble rises it becomes increasingly less able to scavenge particles, consistent with the transition from free-slip to nonslip on the bubble’s surface as it collects surfactant. D) Scaling of *N* accounting for the bursting physics and particle scavenging as a function of $(H/R_{b}{)}^{1/2}\chi \frac{4}{3}\pi r_{d}^{3}$ (Eq. 3), with $r_{\mathrm{MP}}/r_{d}$ color coded. The solid line is a fit with dimensionless prefactor of 4.5. The inset shows $E=N/(\chi \frac{4}{3}\pi r_{d}^{3})$ as a function of $(H/R_{b}{)}^{1/2}$ as well as the data from (35) [reprinted with permission from Ref. (35). Copyright 2023 American Chemical Society]. As in C, each set of experiments varying only *H* (data with a consistent color) shows the $E\propto (H/R_{b}{)}^{1/2}$ scaling.

Figure 4A shows the number of particles per drop, *N*, versus the first dimensionless group, $r_{\mathrm{MP}}/r_{d}$ at constant H=1.5 cm. The liquids used include deionized (DI) water, ethanol–water mixtures, and salt water with various microplastic sizes and bubble sizes. The data are colored by $\chi 4\pi r_{d}^{3}/3$, the second dimensionless group. The average number of particles transported ranges from about 0.1 to 300. While $O(10^{2})$ particles can be captured for $r_{\mathrm{MP}}/r_{d}\ll 1$, the vertical extent of the data with decreasing χ (and the vertical striation by color) indicates that *N* is a stronger function of $\chi 4\pi r_{d}^{3}/3$. $r_{\mathrm{MP}}/r_{d}$ describes a cutoff as $r_{\mathrm{MP}}/r_{d}\approx 1$ above which no particles are transported via jet drops: the size of the jet drop being the maximum particle size that can be transported [consistent with results of other studies (7)]. Bubbles are created in salt water, DI water and ethanol–water mixtures to test the influence of the liquid properties on $r_{d}$ and the number of microplastics particles emitted, and we demonstrate a universal behavior in the emissions of microplastic by a bursting bubble jet drop (Figs. 3 and 4).

The maximum possible number of particles that can be transported is found by replacing the total volume of liquid in the jet drop by densely packed spheres of size $r_{\mathrm{MP}}$, yielding $N_{\max}=\frac{\pi }{3\sqrt{2}}(\frac{r_{\mathrm{MP}}}{r_{d}}{)}^{-3}$, where $\pi /(3\sqrt{2})$ is Carl Gauss’s dense-packing coefficient for spheres (52), shown by the gray dashed line in Fig. 4A, and all of the data lies well beneath it.

Assuming that the particles are well mixed, any volume of liquid, *V*, contains an average of χV randomly dispersed particles (53). As such, the expected number of microplastic pieces per jet drop can be written as


(2) $$N=E\chi \frac{4}{3}\pi r_{d}^{3},$$

where *E* is defined as an efficiency or enrichment factor (35, 36). It physically represents how much more concentrated the particles are in the jet drop compared to the bulk liquid. Figure 4B shows *N* as a function of $\chi \frac{4}{3}\pi r_{d}^{3}$ for the same data as in A, with the data color coded by $r_{\mathrm{MP}}/r_{d}$. The data are shown to be well described by Eq. 2 as the black line shows. At the constant value of H=1.5 cm, the efficiency factor is well approximated as a constant value of $E=E_{c}\approx 11$ obtained by least square fit. The strong effect of changes in χ can be seen for the data with the smallest values of $r_{\mathrm{MP}}/r_{d}$ where a large range of χ spreads the data over almost all values of *N* observed.

### Particle scavenging

After analyzing microplastic transport at a constant rise height, bubbles are released from increasing depth (from 1.5 to 80 cm) to study their scavenging of particles. The effect of increasing *H* is shown in Fig. 4C. Four different particle-drop-bubble size combinations are shown (see colorbar). Only the needle depth was changed across each set of points with a consistent color, and the vertical spread of the data is due to variations in $r_{d}$ and χ. Figure 4C shows that the number of particles *N* increases with *H* and can be described by a $(H/R_{b}{)}^{1/2}$ scaling.

The dashed line represents the $N\propto H/R_{b}$ scaling from the interceptor model (41) which assumes that the surface is completely mobile and captures particles at a constant rate. Nonconstant rate of particle collection as the bubble rises has been previously reported (18, 43, 44). The $N\propto (H/R_{b}{)}^{1/2}$ scaling suggests that when considering large variations of $H/R_{b}$, the bubble’s surface looses its free-slip condition as it scavenges microplastic particles and surface-active molecules (43), transitioning to a nonslip condition (54–57). Indeed the collection rate for a sphere with a nonslip surface is always less than that of one with a free-slip surface for a fixed particle and bubble size (43, 58), which provides a rationale for the observed $N\propto (H/R_{b}{)}^{1/2}$ scaling.

Finally, Fig. 4D shows the final scaling for *N* accounting for both the scavenging ($E=E_{0}(H/R_{b}{)}^{1/2}$) and surface-bursting (Eq. 2), leading to


(3) $$N=E_{0}\chi \frac{4}{3}\pi r_{d}^{3}{\left(\frac{H}{R_{b}}\right)}^{1/2},$$

where $E_{0}$ is a dimensionless prefactor found to be $E_{0}\approx 4.5$ by best fit shown by the solid line. The inset shows $E=N/(\chi \pi r_{d}^{3}4/3)$ as a function of $(H/R_{b}{)}^{1/2}$ and includes data from Ref. (35). The $E\propto (H/R_{b}{)}^{1/2}$ scaling is clear for groups of consistent colors. While correction factors for *E* have been proposed accounting for shape variation of the particles (35) and transfer from the bubble to the jet drop (36), we show that the the average number of microplastics per jet drop can be described by a simple model only accounting for the bursting and scavenging processes.

## Modeling global emission of oceanic microplastic

Having elucidated and quantified the microplastic emissions by individual bubble bursting, we now aim to quantify microplastics emissions at the ocean surface. For drop production at the ocean surface, we leverage the mechanistic approach proposed by Ref. (20) that specifically introduces a jet drop emission function $F_{d}(r_{d})$, coherent with sea spray aerosols field observations and previously proposed sea spray generation functions (17, 19, 21, 60, 61). The jet drop emission function provides the size distribution per unit ocean area per unit time and is sensitive to wind, waves and sea surface temperature (20). With data of the microplastic concentration at the surface of the ocean, $\alpha_{\mathrm{MP}}$ (mass concentration per unit ocean surface area) (8), the oceanic microplastic emission by bubble bursting can be written as


(4) $$S_{\mathrm{MP}}\;=\int F_{d}(r_{d})\frac{\alpha_{\mathrm{MP}}}{z_{\mathrm{MP}}}E(H,R_{b})\frac{4\pi }{3}r_{d}^{3}\;dr_{d},$$

which has units of mass of microplastic per unit area of ocean per unit time. The ratio $\alpha_{\mathrm{MP}}/z_{\mathrm{MP}}$ represents the mass concentration per unit volume over a well mixed microplastic layer of depth $z_{\mathrm{MP}}$. As shown in Fig. 4D, $E=E_{0}(H/R_{b}{)}^{1/2}$, and since $r_{d}$ is a unique function of $R_{b}$ for a given $l_{\mu }$ (47) (see Fig. 3), the efficiency factor can be represented as a function of *H* and $r_{d}$: $E(H,R_{b})\equiv E(H,r_{d})=E_{1}[H/r_{d}(R_{b}/l_{\mu }){]}^{1/2}$, with $E_{1}\approx 1.7$ fitted to the data (Fig. S7 in the online supplementary material).

Bubbles on the ocean are created by breaking waves as air is entrained under the surface. The characteristic depth of bubble entrainment has been found to scale with the significant wave height, $H_{s}$ (62–65) so that we consider the height over which the bubbles are rising $H=H_{s}/2$. While there are multiple mechanisms which disperse microplastic in the ocean (66), the turbulent velocity field created by breaking waves—which entrain air to create bubbles—extends with the significant wave height (67), so we consider that the microplastic layer can be written as $z_{\mathrm{MP}}=H_{s}/2$. This is consistent with microplastic transport models which have a vertical length scale proportional to wave height (11, 68, 69). With these assumptions on *H* and $z_{\mathrm{MP}}$, Eq. 4 becomes


(5) $$S_{\mathrm{MP}}=\zeta \int F_{d}(r_{d})\alpha_{\mathrm{MP}}{\left(\frac{1}{H_{s}r_{d}}\right)}^{1/2}\frac{4\pi }{3}r_{d}^{3}\;dr_{d},$$

where ζ≈2.4 is the combined prefactor from the efficiency function, depth of entrainment and microplastic layer. Because the scavenging rate is found to decrease with bubble rise height, the total amount of microplastic ejected depends on the ocean conditions through both $F_{d}$ and the bubble scavenging.

**Fig. 5.**
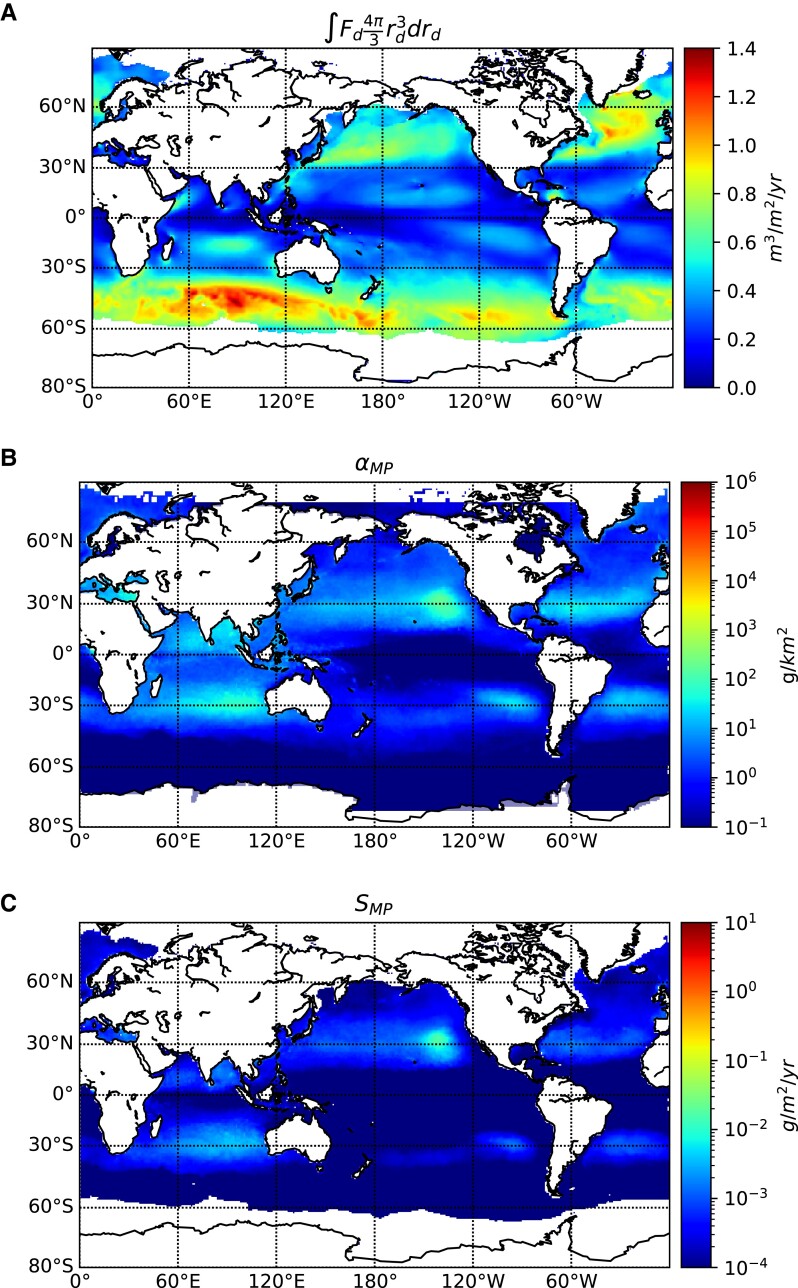
Global microplastic ejection from the ocean. Terms in Eq. 4 are illustrated, as yearly average. A) Volume of liquid contained in jet drops ejected from the ocean by bursting bubbles per unit area per unit time in the ocean, constrained by surface wind and waves following Ref. (20), for the year of 2014. The strong production at high latitude corresponds to high winds and waves. B) Microplastic mass concentration within 5 m of the ocean surface and ranging in size from $100\;\mu m<2r_{\mathrm{MP}}<800\;\mu m$ in 2020 from Ref. (59) C) Microplastic emissions following Eq. 5 using the microplastic distribution shown in panel B. Integrating over the globe, the emission is ≈ 0.1 Mt/yr.

The number and mass of microplastic particles emitted can be evaluated using the sea spray generation function (20), ocean-surface microplastic concentration (8), and estimates of the global sea state. Figure 5A shows the annual mean of the jet drop emission function [expressed in drop volume emitted per year per ocean surface area, computed for a representative year (2014) using realistic wind forcing, see online supplementary material and Ref. (20)] with strong production at high latitude corresponding to high winds. Figure 5B shows the concentration of “ejectable” microplastic estimated from Ref. (59). Particles ranging in size from $100\;\mu m<2r_{\mathrm{MP}}<800\;\mu m$ within 5 m of the ocean’s surface in 2020 are shown (expressed in mass per ocean surface area). Figure 5C shows the total amount of microplastic ejected out of the ocean accounting for the wind and waves through the sea spray generation function (Figure 5A) and the microplastic concentration map (Figure 5B) shown to produce an annual ocean microplastic emission map. High emissions near the coast are the result of the high concentration of microplastic there. The annual emission is then obtained by global integration. Considering various possible microplastic concentration maps leads to an annual emission range of 0.02 to 7.4 Mt/yr with a best guess estimate of 0.1 Mt/yr.

### Caveats and limitations

As shown in Fig. 4, Eq. 3 describes well the amount of microplastic ejected by jet drops across a wide range of microplastic concentrations, bubble sizes, and rise distance. The uncertainties in the bursting physics is well constrained in the laboratory as shown in Fig. 4, while upscaling uncertainties are estimated to be within a factor of 2 to 4 even considering the complex multi-scale processes (20). However, the dominant uncertainty of our global emission estimate is the limited knowledge for ocean microplastic concentration; it is both scarce in temporal and spatial coverage and has a coarse size resolution (3, 8). Furthermore, they are interpolated from limited data sets across multiple years standardized in time to what would be observed in 2014 from Ref. (8), or 2020 in Ref. (59). Table 1 outlines the sensitivity of the global emission to the choice of microplastic concentration coverage map. An upper-limit of concentration is provided by Ref. (8) which bins together particles sizing from 330μm to 200 mm in diameter. The average particle radius in any given area however is 1±0.5mm, which indicates that a considerable portion of particles are of an “ejectable” size, and with it the annual emission rate for 2014 is found to be 7.4 Mt. To estimate only the ejectable amount of microplastic from this dataset, a size distribution $P(r_{\mathrm{MP}})$ must be assumed (see methods) and be limited to 1 mm.

**Table 1.** Sensitivity of global ocean microplastic emission calculated by Eq. 5 to the microplastic concentration map employed ($\alpha_{\mathrm{MP}}$).

Microplastic concentration map	van Sebille et al. (8) (all data)	van Sebille et al. (8) (1 mm cutoff)	van Sebille et al. (8) (1 mm cutoff)	Brahney et al. (5)	Kaandorp et al. (59)
$\min(2r_{\mathrm{MP}}$ )	330μm	330μm	330μm	0.3μm	100μm
$\max(2r_{\mathrm{MP}})$	200 mm	1 mm	1 mm	70μm	800μm
Assumed $P(r_{\mathrm{MP}})$		$P(r_{\mathrm{MP}})=Ae^{-r_{\mathrm{MP}}/\langle r_{\mathrm{MP}}\rangle }$	$P(r_{\mathrm{MP}})=\left\{\begin{array}{cc} B, & r_{\mathrm{MP}}<\langle r_{\mathrm{MP}}\rangle \\ C, & r_{\mathrm{MP}}>\langle r_{\mathrm{MP}}\rangle \end{array}\right\}$		
Global emission	7.4 Mt/yr	0.06 Mt/yr	0.1 Mt/yr	0.02 Mt/yr	0.1 Mt/yr

Four separate concentration maps are employed, and the global emission of each is presented. Maps from Ref. (8) bin together microplastic from $2r_{\mathrm{MP}}=330\;\mu m$ to 200 mm, with the upper end out of the range that can be ejected by jet drops (as the maximum jet drop diameter is about 1 mm). As such, the 7.4 Mt/yr emission rate obtained from the total mass is an upper bound. Assuming an exponential size distribution of that dataset which preserves the total number of plastic at each location and integrating from $330\;\mu m<2r_{\mathrm{MP}}<1\;\mathrm{mm}$ gives an annual emission rate of 0.06 Mt/yr (see Figs. S18 and S20 in the online supplementary material). Alternatively, by assuming a piecewise distribution of two constant values on either side of the mean particle size that preserves both the total number and total mass at each point, global emission is estimated to be 0.1 Mt/yr (see Figs. S19 and S21 in the online supplementary material). Using the microplastic dataset from Ref. (5), which has a size cutoff of 70μm, significantly lower than the largest size that can be emitted, yields global emissions of 0.02 Mt/yr (see Figs. S11 and S16 in the online supplementary material). Using the dataset from Ref. (59), microplastic within 5 m of the ocean’s surface ranging in size from $100\;\mu m<2r_{\mathrm{MP}}<800\;\mu m$ in 2020 is estimated to be ejected at a rate of 0.1 Mt/yr. This emission rate agrees well with that of the piecewise size distribution of the dataset from Ref. (8).

We test two different size distributions, an exponential and a piecewise distribution which both preserve the total number for every datum of the original dataset from Ref. (8) integrating over the full range of sizes (see methods). Once determined, the distributions are then integrated within the range of ejectable microplastics from $330\;\mu m<2r_{\mathrm{MP}}<1\;\mathrm{mm}$ to describe the amount of ejectable microplastics. The global emission assuming the exponential and piecewise size distributions are shown in Table 1 to be 0.06 Mt/yr and 0.1 Mt/yr respectively; demonstrating a high sensitivity to the choice of distribution and size cutoff of microplastics. We note that in all these calculations, the drop diameter being emitted is the same, going up to 1 mm, and argue that the large drops ejected are essential to accurately estimating the mass of microplastic emissions. Using the recent dataset from Ref. (59) and an upper size of 800 microns leads to emission of 0.1 Mt/yr.

Some works calculating microplastic emissions chose to assume a constant microplastic concentration across all oceans (70). Others (7) employed a concentration distribution from Ref. (5) which features a maximum particle size of 70μm and was calibrated with Ref. (8). Using the coverage proposed by Ref. (5) in Eq. 4, we find a microplastic emission rate of 0.02 Mt/yr, significantly lower due to the maximum size of 70μm considered by Ref. (5) which is well below the maximum particle size able to be transported by jet drops (≈500μm). The sensitivity of the global emission to the concentration map motivates improved concentration and size-distribution measurements of microplastic ranging in size from $50\;\mu m<r_{\mathrm{MP}}<500\;\mu m$.

While we demonstrate in this work that microplastic can be ejected if it is the same size or smaller than the jet drops (which have a maximum size of $2r_{\mathrm{MP}}\approx 1\;\mathrm{mm}$ for sea water), our model does not describe their subsequent transport into the turbulent atmospheric boundary layer. Even without microplastic, a large volume of liquid droplets redeposit to the ocean after being ejected to the region just above the surface: see Fig. 5 of Ref. (19). As such, our model describes the emission of microplastic from the ocean surface, not the flux that is actually transported to the upper atmosphere.

Another limitation of the global emission function is that the microplastic concentration data used is a constant annualized mean for each spatial position. The emission function (and sea state) however are calculated on a much shorter timescale of *O* (hours). As such, nonlinearities between temporal variations in the microplastic concentration and jet drop production flux could affect the final result due to this difference in timescale sampling.

## Conclusion

The amount of microplastic ejected from the ocean is determined by studying individual drops created by bursting bubbles to create a global emission model which depends on local sea state. We demonstrate experimentally by direct high-speed video visualization and measurement that microplastic particles (with diameters from 10 to 280μm) are transported out of a bulk liquid such as the ocean and into the air above by jet drops resulting from bursting bubbles. The size of jet drops containing microplastics is found to be well described by theoretical scaling laws derived for drops without microplastics. The number concentration of microplastic particles in emitted drops is found to be proportional to the concentration of particles in the bulk and jet drop volume, with an efficiency or enrichment factor $E=E_{0}(H/R_{b}{)}^{1/2}$ (we find $E_{0}\approx 4.5$), suggesting a reduction in surface mobility as the bubbles rise over large $H/R_{b}$. The number of emitted microplastic particles for a single bubble bursting is thus given by $N=E_{0}(H/R_{b}{)}^{1/2}\chi \frac{4\pi }{3}r_{d}^{3}$.

Given that jet drops produced by bubble bursting at the ocean surface dominate sea spray aerosols emissions of size 2μm to 1 mm (17, 20) and that microplastic pieces are carried by jet drops equal to or larger in size, jet drops can be effective at emitting microplastic up to *O* (1 mm) in size and are hence responsible for most of the emitted mass of microplastic from the ocean to the atmosphere. To estimate the global microplastic emissions by the ocean, the individual emission efficiency is combined with a jet drop emission function [from Ref. (20)] and microplastic concentration in the ocean [from Ref. (8), see the Materials and methods section for a discussion of particle size compared to that of the emission function]. Thus, we provide an independent bottom-up estimate of microplastic emissions from the ocean that can be compared to previous estimates obtained from atmospheric observations and inverse modeling (3, 5) and other independent estimates which did not consider any efficiency factor or scavenging dynamics (7). We obtain a range of annual microplastic ocean emission from 0.02 to 7.4 Mt/yr with the upper bound accounting for sizes larger than what can be ejected by jet drops. The upper bound is similar in magnitude to the best guess value of 8.6 Mt/yr from Ref. (5). Our best guess using the most recent concentration data set from Ref. (59) gives an estimate of 0.1 Mt/yr. The emission employing the microplastic maps of Ref. (5) are much larger than those from Refs. (7, 70) as we account for larger droplets being emitted. While the upper bound of our emission estimate supports the hypothesis that ocean emission of microplastic plays a significant role in global-scale microplastic transport as proposed by Ref. (5), the precise emission remains difficult to estimate better without improved concentration maps that include accurate size distributions.

Our study provides an independent estimate of the microplastic emission by the ocean and reduces the associated uncertainties by identifying and quantifying the leading emission mechanism. As a consequence, the remaining large part of the uncertainty comes from the limited oceanic observations of microplastic concentration and the lack of high resolution size distributions of microplastic pieces present at the surface of the ocean (particularly at scales of less than 50μm) confirming previous assessments (3, 5). A significant effort in observational data of marine microplastic data is necessary to reduce these present uncertainties.

## Materials and methods

### Experimental methods

We analyze the ejection dynamics visually, using two high-speed cameras, a phantom V2012 and a phantom 4 K, to capture videos (up to 22,000 frames per second) of a single bubble bursting. An example of bubble bursting in water is shown in Fig. 2. At each experimental condition, approximately 10 videos are taken of the bursting event from both above and below the free surface.

The radius of the bubble, $R_{b}$, is varied by employing different sized needles; inner diameters ranging from 0.4 to 2.7mm to produce bubbles of size $R_{b}=0.88$ to 1.90 mm by slowly pushing air through the needle with a syringe pump so that only a single bubble is created at a time. Two different liquid containers were employed. For the H=1.5 cm conditions, a shallow dish of size 2×10×10cm was used to hold the liquid, while the H>1.5 cm trials were conducted in a tank of size 80×20×20cm. In both containers, the bubbles did not touch the bottom or sides during the rising or bursting process.

In each trial, spherical microplastic particles ranging in radius, $r_{\mathrm{MP}}$, from 5 to 140μm were added to the liquid. All particles were polyethylene with a density of $1.00\;g/{\mathrm{cm}}^{3}$ except for one set of trials with $r_{\mathrm{MP}}=5\;\mu m$ hollow borosilicate glass with a density of $1.1\;g/{\mathrm{cm}}^{3}$. We have considered microplastic of near neutral density compared to the liquid, with slight buoyancy variations, the density ratio $\rho_{\mathrm{MP}}/\rho_{l}$ varying from 0.97 to 1.14 (see Table S2 of the online supplementary material). We do not observe any significant change due to density in the transport over this range of density variation. The concentration of particles added ranges from $\chi =1.02\times 10^{8}$ to $1.166\times 10^{11}$ or equivalently $\alpha =0.1\;{\mathrm{gm}}^{-3}$ to $1166\;{\mathrm{gm}}^{-3}$; $[\chi ]=L^{-3}$ is a number concentration of pieces per unit volume of liquid while $[\alpha ]=ML^{-3}$ is the sum of microplastic mass per unit volume of liquid. During measurements, the mixture is mixed routinely to ensure that χ is constant for the duration of each trial. The size of the tank is much larger (at least a factor of 10${}^{6}$) than the volume of jet drops, so the drop ejection does not change χ over the duration of each experiment.

While only spherical particles were used in this study, the emission of other shapes has been studied previously. Some works find that emission does not vary significantly between spheres and fibers (71), and others find that fibers are not ejected as well (7); particle shape could be an area of future research. This work does not consider the transport of particles at the ocean’s surface which is complex, and has been shown to be a function of both the particle’s shape and the flow field (11, 72–74). This study used both polyethylene and borosilicate glass particles, and no significant difference was observed in the capture and emission between the different materials, which is consistent with other work (7). Biofouling is another effect which was not considered in this work, and it has been shown to change the effective density of microplastic particles (75). As the concentration measurements used in this model are taken in the ocean, they have already accounted for any effect biofouling has on the presence of particles at the ocean’s surface. A recent work (76) showed that there is a correlation between the presence of microplastics and surfactant concentrations in the ocean; this could be due to biofouling which would have the potential to alter the liquid properties (such as surface tension).

Multiple liquids were used in the present study: DI water, salt water with a salinity of 42 g/kg similar to sea water (“Sea Salt” ASTM D1141-98), and ethanol–water solution (20% by mass). A small amount of sodium dodecyl sulfate (SDS) is added to the liquid at a concentration of 6.6μm which is several orders of magnitude below the critical micelle concentration of SDS of 8.2mM. As a surface-active (surfactant) chemical, SDS is able to provide surface forces and is a common additive to weakly contaminate the surface—making it more like the marine environment. In this concentration range, the difference in surface tension due to the addition of surfactant is within measurement error using the pendant drop method (77). Liquid properties (density, viscosity, and surface tension) are provided in Table S1 of the online supplementary material. Varying the liquid properties enables us to understand the role of the physico-chemical properties and derive universal scalings that can be applied to sea water or any other liquid.

Together with the direct images obtained by high-speed videos (see Fig. S1 of the online supplementary material for a diagram of the experimental setup), the jet drops produced are also captured to count the number of microplastic particles they contain. A flat petri dish is positioned above the free surface: ranging from 1 to 6 cm above the surface so that the jet drops impact at a low enough velocity to stick. The drops are allowed to dry, leaving behind the microplastic that was captured by the jet drop. The deposited particles are counted using a Leica DMI4000 B microscope. For each condition, O(50−−100) drops are collected and the number of microplastic particles in each drop are counted and their average is reported. Examples of the microscope view of particles on the collection plate are shown in Figs. S2 and S3 of the online supplementary material, and examples of microplastic number distributions are shown in Figs. S4–S6 of the online supplementary material.

### Global model parameters and uncertainties

Number and mass coverage of microplastics have been proposed with interpolation models varying by a factor of 2 (shown in Figs. S8–S11 of the online supplementary material) (5, 8), but the uncertainty on the data is probably larger and related to the binning of most microplastic size measurements. The concentration map of particles sized $100\;\mu m<2r_{\mathrm{MP}}<800\;\mu m$ located within 5 m of the ocean’s surface in 2020 from a recent study (59) is also shown in Fig. S12 of the online supplementary material. The uncertainty of the resulting output depending on mass coverage map is shown in Figs. S13–S17 of the online supplementary material. Furthermore, the limited measurements in the ocean are taken in a variety of conditions which affects their spatial distribution in the ocean (9–11). In Ref. (8), the bin size ranged from 0.15 mm $<r_{\mathrm{MP}}<$ 100 mm, and over 90% of the data was collected with a 0.33 mm net mesh. As such, it is treated as an upper bound of microplastic concentration. Other studies [such as Refs. (5, 78)] suggest a somewhat smaller number could also be appropriate. Estimates of ejectable microplastic are determined from Ref. (8) by assuming a particle size distribution. As such, the total number of particles is $\int_{\min(r_{\mathrm{MP}})}^{\max(r_{\mathrm{MP}})}P(r_{\mathrm{MP}})dr_{\mathrm{MP}}$ and the total mass is $\int_{\min(r_{\mathrm{MP}})}^{\max(r_{\mathrm{MP}})}P(r_{\mathrm{MP}})\frac{4\pi }{3}\rho_{\mathrm{MP}}r_{\mathrm{MP}}^{3}dr_{\mathrm{MP}}$ for any given latitude and longitude. The full range of sizes from Ref. (8) goes from $330\;\mu m<2r_{\mathrm{MP}}<200\;\mathrm{mm}$. We consider two options for the distribution in order to account for the fact that particles above 1 mm are not ejected by jet drops. The distributions we consider are an exponential $P(r_{\mathrm{MP}})=Ae^{-r_{\mathrm{MP}}/\langle r_{\mathrm{MP}}\rangle }$ (where $\left\langle r_{\mathrm{MP}}\right\rangle$ is the average size for each datum) and a piecewise distribution, $P(r_{\mathrm{MP}})=\{B$ for $r_{\mathrm{MP}}<\langle r_{\mathrm{MP}}\rangle$ and *C* for $r_{\mathrm{MP}}>\langle r_{\mathrm{MP}}\rangle \}$, where *A*, *B*, and *C* are determined at each point. Concentration maps of each assumption are shown in Figs. S18 and S19 of the online supplementary material, respectively. The piecewise distribution maintains both the mass and number for each datum. Once determined, the distributions are then integrated from $330\;\mu m<2r_{\mathrm{MP}}<1\;\mathrm{mm}$ to describe the amount of ejectable microplastic, and summarized in Table 1. The ejection rate for each size distribution is shown in Fig. S20 and S21 of the online supplementary material, respectively. A comparison between the concentration and subsequent emission assuming a piecewise distribution of the data in (8) and the most recently published concentration dataset in Ref. (59) is shown in Fig. S22 of the online supplementary material. While the assumptions that produce each are different, the estimated concentration of ejectable microplastic and the subsequent emission maps are strikingly similar and the total annual emission of each is 0.1 Mt/yr.

Global emission is also computed with the dataset from (5) which is treated as a lower bound due to its relatively small maximum particle size of 70μm. Measurements (79) of oceanic plastic with detailed size distinctions below 330μm found that particle number concentration was approximately constant for all size bins between 50 and >1,000μm, and as much as 50% of the number of microplastic particles in the ocean are smaller than 50μm. Another study (74) focusing on microplastic concentration from the surface to 10 m of depth found that the total amount of microplastic near the ocean’s surface may be 10 times larger than what would be estimated by a surface net alone as most ocean-surface microplastic studies use. While precise (both in spatial resolution and the size-binning resolution) data for the size-range necessary are lacking, we have estimated the amount of ejectable microplastic from Ref. (8) and find a range spanning about two orders of magnitude that depends solely on the microplastic concentration coverage chosen.

Microplastic concentration data are reported as a number per unit ocean surface area. The raw data was collected at the ocean’s surface and standardized to account for mixing due to ocean conditions (8). The depth over which this microplastic is spread, $z_{\mathrm{MP}}$ needs to be estimated in our emission model. Following measurements and modeling of ocean-surface microplastic transport, we assume that the characteristic depth scales with significant wave height to obtain the volumetric concentration $\alpha_{\mathrm{MP}}/z_{\mathrm{MP}}$. However, the relationship between $z_{\mathrm{MP}}$ and ocean conditions remains an open question as there are very limited observations of underwater microplastic concentration profiles (66, 68, 69, 79), and there is some degree of uncertainty with this estimate. The prefactor by which $z_{\mathrm{MP}}$ and *H* scale with significant wave height is estimated to range from 0.25 to 0.75. The uncertainty of the prefactor of the jet drop emission function is estimated to be a factor of 2 in Ref. (20). These uncertainties of the emission process are estimated to be less significant than the uncertainties of the microplastic concentration data.

## Supplementary Material

pgad296_Supplementary_Data

## Data Availability

The microplastic abundance dataset published in Ref. (59) can be found at: https://doi.org/10.24416/UU01-LDAGQN. The data taken in this study are shown in Figs. 3–5 is available at: https://doi.org/10.34770/79v7-0v27.
