# Supplementary material for: Ocean emission of microplastic
Source: PNAS Nexus. 2023 Oct 3;2(10):pgad296. doi: 10.1093/pnasnexus/pgad296 (PMC10547021; doi:10.1093/pnasnexus/pgad296)
Supplement: pgad296_Supplementary_Data [file pgad296_supplementary_data.pdf]

Supporting Information for "Ocean emission of microplastic"

Daniel B. Shaw

Department of Mechanical and Aerospace Engineering,
Princeton University, Princeton NJ 08540

Qi Li

School of Civil and Environmental Engineering,
Cornell University, Ithaca NY 14853

Janine K. Nunes

Department of Mechanical and Aerospace Engineering,
Princeton University, Princeton NJ 08540 and
Department of Chemical and Biological Engineering,
Princeton University, Princeton NJ 08540

Luc Deike*

Department of Mechanical and Aerospace Engineering,
Princeton University, Princeton NJ 08540 and
High Meadows Environmental Institute,
Princeton University, Princeton NJ 08540l

(Dated: September 6, 2023)

CONTENTS

List of Figures 2

List of Tables 3

Introduction 3

I. Experimental setup: Jet drop capture and microplastic counting 4
- A. Setup 4
- B. Liquid properties 4
- C. Microscope measurements 4
- D. Particle count distribution 4
- E. Colloidal particles 4

II. Background on Film and jet drop production mechanism 5
- A. Film drop production 5
- B. Jet drop production 5

III. Microplastic emission model: jet drop emission function 6
- A. Microplastic concentration maps 7

References 26

LIST OF FIGURES

S1 Experimental setup 14

S2 Microscope image 1 14

S3 Microscope image 1 15

S4 Histogram 1 15

S5 Histogram 2 16

S6 Histogram 3 16

S7 Bursting model: $N = 1.7\chi\frac{4\pi r_d^3}{3}\left(\frac{H}{r_d}\right)^{1/2}$ 17

S8 Maximenko model concentration map 17

* ldeike@princeton.edu

S9 Lebreton model concentration map . . . 18
S10 van Sebille model concentration map . . . 18
S11 Microplastic concentration from Brahney et al. 2021 . . . 19
S12 Kaandorp et al. 2023 concentration map . . . 19
S13 Maximenko model mass flux . . . 20
S14 Lebreton model mass flux . . . 20
S15 van Sebille model mass flux . . . 21
S16 Mass flux of microplastic from Brahney et al. 2021 . . . 21
S17 Mass flux of microplastic with the concentration map from Kaandorp et al. 2023 . . . 22
S18 Ejectable microplastic concentration: $P(r_{MP}) = Ae^{-r_{MP}/\langle r_{MP}\rangle}$ . . . 22
S19 Ejectable microplastic concentration: $P(r_{MP}) = \{B$ for $r_{MP} < \langle r_{MP}\rangle$ and C for $r_{MP} > \langle r_{MP}\rangle\}$ . . . 23
S20 Ejectable microplastic mass flux: $P(r_{MP}) = Ae^{-r_{MP}/\langle r_{MP}\rangle}$ . . . 23
S21 Mass flux of ejectable microplastic assuming $P(r_{MP}) = \{B$ for $r_{MP} < \langle r_{MP}\rangle$ and C for $r_{MP} > \langle r_{MP}\rangle\}$ . . . 24
S22 Comparison of concentration and mass flux of microplastic assuming a piecewise size distribution and the data set from Kaandorp et al. 2023 . . . 25

LIST OF TABLES

S1 Liquid properties. . . . 10
S2 Microplastic particles studied. . . . 11
S3 Experimental trials for $H = 1.5$ cm. . . . 12
S4 Experimental trials for $H > 1.5$ cm. . . . 13

INTRODUCTION

Information about the experimental setup, global model, and background of the relevant bubble bursting physics are provided in this document.

I. EXPERIMENTAL SETUP: JET DROP CAPTURE AND MICROPLASTIC COUNTING

A. Setup

In order to measure the number of particles transported by each jet drop, a plate is held above the bursting bubbles as shown in Figure S1. Its height it set such that the jet drop travels up and impacts the plate with a low enough velocity that it sticks. The collection plate is moved between each subsequent bubble bursting event so that each jet drop is separate. The drops are allowed to dry, leaving behind any microplastic particles that had been transported. The number of particles in each drop is counted in a LEICA DMI4000 B microscope.

B. Liquid properties

Three different liquids were used in this study: DI Water, 20 % (by mass) ethanol-water, and salt water. The properties of the liquid are shown in Table S1.

C. Microscope measurements

An example image of the view from the microscope is shown in Figures S2 and S3.

D. Particle count distribution

The number of microplastic particles captured per bursting event are counted in this study. Example histograms of this data are shown in Figures S4, S5, and S6.

E. Colloidal particles

While a precise definition of a colloid is complex and depends on the context, they typically feature a disperse system of small-scale particles which are generally 1 nm to 1 µm in size. At this size range, the particle has a very small settling velocity compared to the characteristic flow velocity. The settling velocity, v_s, is determined as the balance between

buoyancy and stokes drag: $v_s = V_P \Delta\rho_P g/(6\pi\mu r_P)$, where V_P is the particle volume, $\Delta\rho_P$ is the density difference between the particle and the surrounding fluid, g is gravity, μ is the dynamic viscosity of the fluid, and r_P is the radius of the particle [1].

II. BACKGROUND ON FILM AND JET DROP PRODUCTION MECHANISM

Some general information on film and jet drop production is described.

A. Film drop production

A detailed discussion on film and jet drop production can be found in [2–4]. When a bubble arrives at the free surface, its cap immediately starts to thin. After some amount of time, the cap punctures and subsequently retracts at a constant taylor-culick velocity: $V_{cap} = \sqrt{2\sigma/(\rho_{liq} h)}$ where σ is the liquid-gas surface tension, ρ_{liq} is the liquid density, and h is the film's thickness [5, 6]. As the film retracts, fluid from the sheet collects in a rim. As the rim grows bigger, it destabilizes due to a Rayleigh-Taylor instability as it follows the shape of the bubble's cap, an arc of radius R_{cap}, with wavelength $\lambda = \sqrt{R_{cap} h}$ and timescale $\tau \sim \sqrt{\rho_{liq}(R_{cap} h)^{3/2}/\sigma}$. These perturbations grow and subsequently pinch off to form droplets. Droplet sizes are linked to the film thickness, preventing particles much larger than the film to be ejected in the air.

B. Jet drop production

The relationship between r_d/l_μ and $La = R_b/l_\mu$ has been studied significantly both experimentally and numerically. [7] proposed:

$$\frac{r_d}{l_\mu} \sim \left(\sqrt{\mathrm{La}}\left(\sqrt{\frac{\mathrm{La}}{\mathrm{La}_*}} - 1\right)\right)^{5/4} \tag{1}$$

where $La_* \approx 550$ is the drop ejection threshold (about 5 µm in salt water at $20°C$) [8] based on the intertial-capillary focusing of the capillary waves and balancing forces during the cavity reversal and jet formation. [9] and [10] proposed an alternate scaling law based on

wavelength selection during the cavity collapse combined with an intertial-capillary balance

$$\frac{r_d}{l_\mu} \sim \mathrm{La}\left(1 - \left(\sqrt{\frac{\mathrm{La}_c}{\mathrm{La}}}\right)^{1/4}\right) \tag{2}$$

where $\mathrm{La}_c \approx 1000$. The prefactors of Equations 1 and 2 are obtained by least square fitting to experimental and numerical data and are 0.6 and 0.2 respectively in Figure 3.

A bubble residing at a liquid-air interface has both a cavity under the free surface and a thin-film liquid cap above the free surface. The competition of buoyancy driving the trapped air upwards and capillarity which resists the creation of more surface area is characterized by the Bond number: $\mathrm{Bo} = \Delta\rho g R_b^2/\sigma = (R_b/l_c)^2$, where $\Delta\rho$ is the difference in density between the gas and liquid, σ is the surface tension of the interface, R_b is the radius of the bubble, and $l_c = \sigma/\Delta\rho g$ is the capillary length ($l_c = 2.7$mm for a clean water and air interface at $20°C$). A $R_b/l_c \ll 1$ bubble is almost perfectly spherical and resides predominantly below the free surface with a very small liquid thin-film cap. Conversely, a $R_b \gg 1$ bubble is nearly entirely above the free surface and has a half-sphere liquid cap.

The number of jet drops created from a bursting bubble, n, has also been measured in prior work [8, 11–13], and is found to be a function of R_b/l_μ:

$$n = \Omega\left(\frac{R_b}{l_\mu}\right)^{-1/3} \tag{3}$$

where $\Omega = 145$ is a nondimensional prefactor that is estimated by best-fit to data [8]. As size increases, gravity forces increase relative to capillary forces with the transition occurring at $R_b \approx l_c$. For air bubbles produced in water, only one drop is produced at $\frac{R_b}{l_\mu} = 2 \times 10^5$ and no drops observed for higher values of $\frac{R_b}{l_\mu}$.

III. MICROPLASTIC EMISSION MODEL: JET DROP EMISSION FUNCTION

As described in [14], the emission of jet drops depends on the local sea state: wind, waves, sea surface temperature. The generation function, $F_d(r_d)$ is defined as number of emitted drops per unit surface area, per unit time, per unit bin size [15] and is related to the distribution flux of bubbles under breaking waves (defined as the number per unit ocean surface area per unit time per bin size) $Q(R_b)$ [14, 16]. The emission function is then given by [14]:

$$F_d(r_d) = \int Q(R_b)\frac{n(R_b)}{\langle r_d\rangle(R_b)}p\left(\frac{r_d}{\langle r_d\rangle}\right)\mathrm{d}R_b \tag{4}$$

where $n(R_b)$, $\langle r_d \rangle$ are respectively the number and mean radius of jet drops ejected by bubbles of sizes R_b, and are obtained from scaling relationships depending on the liquid viscosity, density and surface tension and verified against laboratory and numerical simulations of single bubble bursting [7–9, 17, 18]. The distribution $p\left(\frac{r_d}{\langle r_d \rangle}\right)$ is taken as a Gamma distribution [8]. Details of the derivation and data used to validate this approach can be found in [14, 16]. The generation function depends on the local sea state and the breaking wave distribution, which can be modeled by a wave model forced by wind product, as described by [14]. The obtained generation function is then compatible with field observation of sea spray aerosols as demonstrated in [14]. Figure in main paper is obtained using the wave model WAVEWATCHIII [19] and wind product JRA-do [20, 21] run over the year of 2014.

A. Microplastic concentration maps

The global microplastic transport model uses local concentration of microplastic as an input. In this work, an upper bound of microplastic concentration data is taken from [22] which bins together particles of 330 µm to 200mm. As maps of global ocean concentration are determined from a series of discrete measurements, they present results from multiple models for the transport of microplastic due to ocean current that are used to go from the discrete measurements to global concentration maps as shown in Figures S8, S9, and S10. The influence of the other various modeled microplastic maps on the total annual emission of microplastic is shown in Figures S13, S14, and S15.

While a maximum particle diameter of 200 mm is well above the maximum diameter of microplastic able to be ejected, O(1 mm), the average particle radius, $\langle r_{MP} \rangle$, of the dataset - obtained by comparing the total number and total mass reported at every point - is $\langle r_{MP} \rangle \approx 1000 \pm 500$ µm, which indicates that a considerable portion of the particles are of a size able to be ejected. To estimate the concentration of microplastic of an 'ejectable' size, a size distribution of particles $P(r_{MP})$ is assumed. At any given point latitude and longitude, the total number of particles is:

$$\int_{\min(r_{MP})}^{\max(r_{MP})} P(r_{MP}) \mathrm{d}r_{MP} \tag{5}$$

and the total mass is defined as:

$$\int_{\min(r_{MP})}^{\max(r_{MP})} P(r_{MP}) \frac{4\pi}{3} \rho_{MP} r_{MP}^3 \mathrm{d}r_{MP} \tag{6}$$

Assuming a size distribution lets us estimate the amount of ejectable microplastics by integrating up to the maximum particle size able to be captured by jet drops: $r_{MP} = 500$ µm. We choose two different distributions which both preserve the total number of particles and average particle size of each point from [22]. The first is an exponential

$$P(r_{MP}) = Ae^{-r_{MP}/\langle r_{MP}\rangle} \tag{7}$$

(corresponding to a Poisson distribution representing random process), and the second is a piece-wise function:

$$P(r_{MP}) = \left\{ \begin{array}{l} B,\ 2r_{MP} < 1\text{mm} \\ C,\ 2r_{MP} > 1\text{mm} \end{array} \right\} \tag{8}$$

where A, B, and C are constants determined at each global position to preserve the total number and average size. Once $P(r_{MP})$ is determined, the mass and number of microplastic are calculated by equations 6 and 5 respectively with 300 µm and 1 mm as the bounds of integration. By integrating over the surface of the globe, the total number of microplastic can be calculated with the size distribution per unit area $P_a(r_{MP}, lat, lon) = P(r_{MP})/A(lat, lon)$ where A is the local area for each location of the microplastic concentration map:

$$\int\int\int P_a(r_{MP})\ \mathrm{d}r_{MP}\ \mathrm{d}lat\ \mathrm{d}lon \tag{9}$$

and the total mass is similarly defined as:

$$\int\int\int P_a(r_{MP})\frac{4\pi}{3}\rho_{MP}r_{MP}^3\ \mathrm{d}r_{MP}\ \mathrm{d}lat\ \mathrm{d}lon \tag{10}$$

The dataset from [23] is also used as input to the global emission model, but the maximum particle size it reports is 70 µm, which is less than the maximum particle size able to be ejected by jet drops ($2r_{MP} = 1$ mm). As such, it is not surprising that its global emission is smaller than that of the original dataset from [22] or those which estimate the ejectable amount of microplastic. This is particularly true for reported mass budgets as these weight the larger particles higher than smaller ones.

The concentration map of ejectable microplastic assuming an exponential and piece-wise size distribution of the dataset from [22] is shown in Figure S18 and S19 respectively. As expected, both show a reduction in the amount of mass that is able to be ejected compared to the original dataset from [22]. The concentration of microplastic from [23] is shown in Figure S11, and as expected it is smaller than the other datasets presented. We also visually

compared the concentration maps from [24] for the relevant particle size range, and we expect a global emission to be similar to that of the concentration map from [23].

The global emission is calculated with the same emission function and sea state (for the year of 2014) for the exponential particle distribution, piecewise particle size distribution, and dataset from [23] are shown in Figures S20, S21, and S16 respectively.

**Liquid**	ρ_l [**kg m^{-3}**]	μ_l [**mPa s**]	σ [**kg s^{-2}**]
DI Water	1000	0.95	0.072
20 % Ethanol	965	1.77	0.040
Salt Water (42.4g / kg)	1024	1.02	0.074

TABLE S1: Liquid properties.

**Particle**	**Liquid**	**Bo**	χ $[m^{-3}]$	α $[gm^{-3}]$	Φ	r_d [mm]
10 µm<br>1.1 g/cm^3	DI W & SDS<br>Ethanol (20% wt)	0.11 - 0.50	$1.74 \times 10^8 - 1.16 \times 10^{11}$	0.1 - 66.6	$9.1 \times 10^{-8} - 6.1 \times 10^{-5}$	0.16 - 0.39
25 µm<br>1.02 g/cm^3	DI W & SDS<br>Salt Water & SDS<br>Ethanol (20% wt)	0.13 - 0.49	1.20×10^{10}	100	9.80×10^{-5}	0.16 - 0.43
45-53 µm<br>1.00 g/cm^3	DI W & SDS<br>Ethanol (20% wt)	0.2 - 0.48	1.02×10^9	66.6	6.66×10^{-5}	0.17 - 0.37
90-106 µm<br>1.00 g/cm^3	DI W & SDS<br>Ethanol (20% wt)	0.40 - 0.48	3.38×10^7	166.6	1.66×10^{-4}	0.17 - 0.38
280 µm<br>1.00 g/cm^3	DI W & SDS	0.48	1.02×10^8	1166.6	1.16×10^{-3}	0.38

TABLE S2: Microplastic particles studied.

$2\ r_{MP}$ [µm]	Liquid	χ [m^{-3}]	# Trials
10	DI W & SDS	1.16×10^{11}	108
10	DI W & SDS	5.79×10^{10}	99
10	DI W & SDS	5.79×10^{10}	99
10	DI W & SDS	2.89×10^{10}	101
10	DI W & SDS	2.89×10^{10}	103
10	Ethanol (20% wt) & SDS	5.79×10^{10}	97
10	DI W & SDS	1.16×10^{11}	100
10	DI W & SDS	1.16×10^{11}	96
10	DI W & SDS	5.79×10^{10}	102
10	DI W & SDS	5.79×10^{10}	98
10	DI W & SDS	2.89×10^{10}	98
10	DI W & SDS	2.89×10^{10}	95
10	DI W & SDS	1.16×10^{11}	118
10	DI W & SDS	1.16×10^{11}	95
10	DI W & SDS	1.74×10^{8}	102
25	DI W & SDS	1.2×10^{10}	90
25	DI W & SDS	1.2×10^{10}	100
25	DI W & SDS	1.2×10^{10}	97
25	DI W & SDS	1.2×10^{10}	102
25	Ethanol (20% wt) & SDS	1.2×10^{10}	100
25	Salt Water & SDS	1.2×10^{10}	79
25	Salt Water & SDS	1.2×10^{10}	103
25	Salt Water & SDS	1.2×10^{10}	54
50	DI W & SDS	1.02×10^{9}	100
50	DI W & SDS	1.02×10^{9}	104
50	Ethanol (20% wt) & SDS	1.02×10^{9}	103
98	DI W & SDS	3.38×10^{8}	93
98	Ethanol (20% wt) & SDS	3.38×10^{8}	104
280	DI W & SDS	1.02×10^{8}	107

TABLE S3: Experimental trials for $H = 1.5$ cm.

$2\ r_{MP}$ [µm]	Liquid	$\chi\ \left[\mathrm{m}^{-3}\right]$	H [m]	# Trials
25	DI W & SDS	2.99×10^9	0.5	19
25	DI W & SDS	2.99×10^9	0.7	16
25	DI W & SDS	2.99×10^9	0.2	17
25	DI W & SDS	2.99×10^9	0.1	17
25	DI W & SDS	2.99×10^9	0.05	17
25	DI W & SDS	2.99×10^9	0.02	17
98	DI W & SDS	3.38×10^8	0.02	76
98	DI W & SDS	3.38×10^8	0.7	58
98	DI W & SDS	3.38×10^8	0.5	62
98	DI W & SDS	3.38×10^8	0.2	63
98	DI W & SDS	3.38×10^8	0.1	64
98	DI W & SDS	3.38×10^8	0.05	60
98	DI W & SDS	3.38×10^8	0.02	54
98	DI W & SDS	3.38×10^8	0.05	58
98	DI W & SDS	3.38×10^8	0.1	53
98	DI W & SDS	3.38×10^8	0.5	56
98	DI W & SDS	3.38×10^8	0.7	54
98	DI W & SDS	3.38×10^8	0.7	56
98	DI W & SDS	3.38×10^8	0.5	51
98	DI W & SDS	3.38×10^8	0.1	50

TABLE S4: Experimental trials for $H > 1.5$ cm.

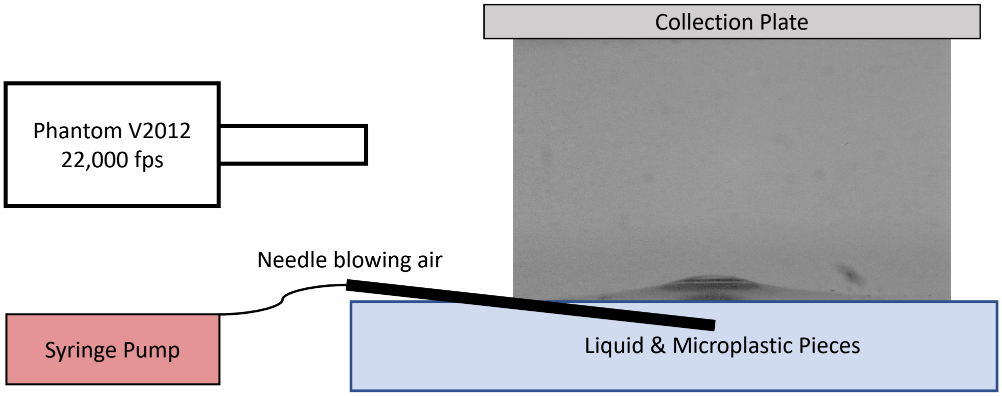

FIG. S1: The experimental setup used to capture jet drops from bursting bubbles. The height of the collection plate is varied depending on the speed of the jet drop ejection to ensure that the drop sticks and does not bounce off. For the trials with a fixed height of $H = 1.5$ cm, the size of the liquid container is 2 cm x 10 cm x 10 cm. For the $H > 1.5$ cm trials, the tank dimensions are 80 cm x 20 cm x 20 cm. In both containers, the bubbles did not touch the bottom or sides during the rising or bursting process.

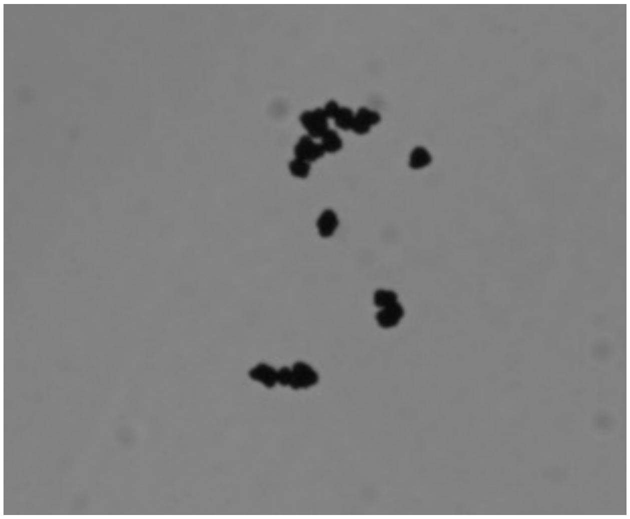

FIG. S2: View of the microplastic pieces that were captured by a jet drop and allowed to dry on the collection plate. The particles shown have a diameter of 25 µm ($\chi = 1.2 \times 10^{10}$ m^{-3}). The lighter semi-transparent specs in the image are artifacts from the microscope while the solid dark pieces are the microplastics. Figure S3 is at the same condition, their difference is due to statistical variation of the process.

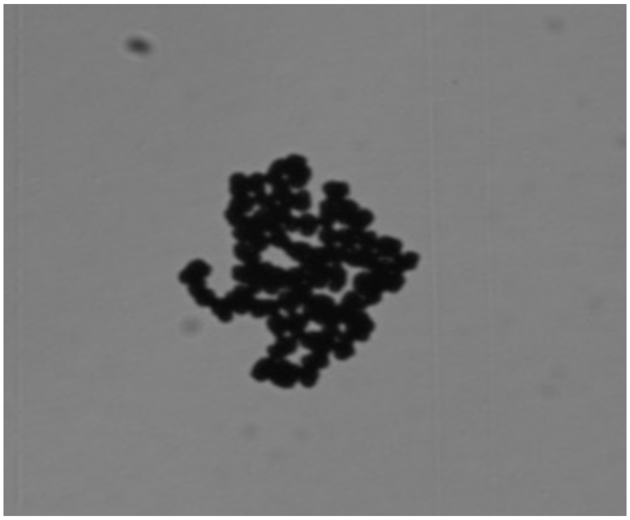

FIG. S3: View of the microplastic pieces that were captured by a jet drop and allowed to dry on the collection plate. The particles shown have a diameter of 25 µm ($\chi = 1.2 \times 10^{10}$ m^{-3}). The lighter semi-transparent specs in the image are artifacts from the microscope while the solid dark pieces are the microplastics. Figure S2 is at the same condition, their difference is due to statistical variation of the process.

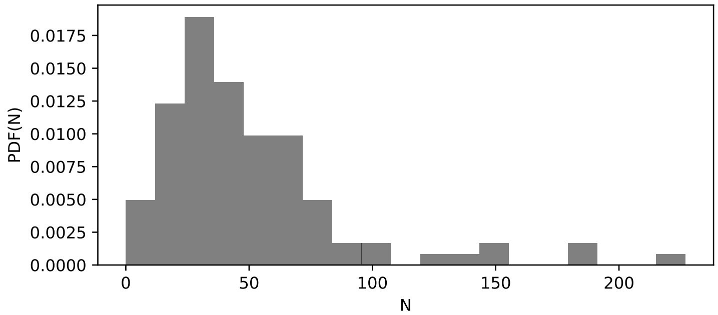

FIG. S4: The results of 102 trials are shown. The experimental conditions are: DI Water, 10 µm microplastic particles, $\chi = 5.79 \times 10^{10}$ m^{-3}, the jet drop diameter is 699 µm, and the bubble radius is 1.9 mm. The average value is $N = 50$.

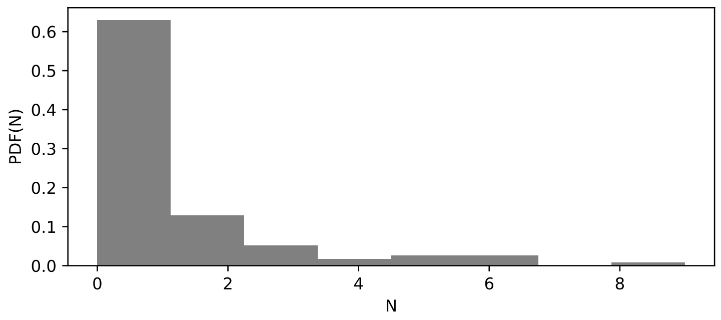

FIG. S5: The results of 103 trials are shown. The experimental conditions are: ethanol, 50 µm microplastic particles, $\chi = 1.02 \times 10^9 \mathrm{m}^{-3}$, the jet drop diameter is 334 µm, and the bubble radius is 1.4 mm. The average value is $N = 1.1$.

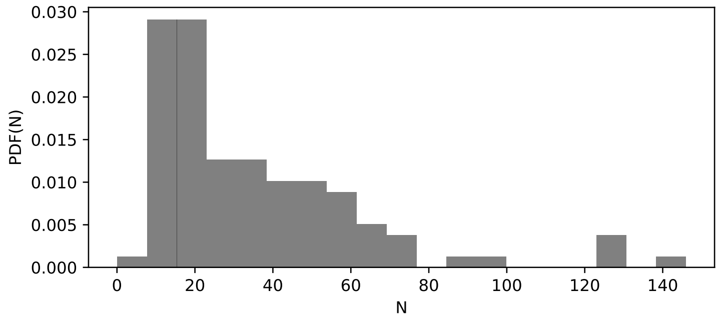

FIG. S6: The results of 103 trials are shown. The experimental conditions are: salt water (salinity of 42.4g / kg), 25 µm microplastic particles, $\chi = 1.20 \times 10^{10} \mathrm{m}^{-3}$, the jet drop diameter is 652 µm, and the bubble radius is 1.7 mm. The average value is $N = 35$.

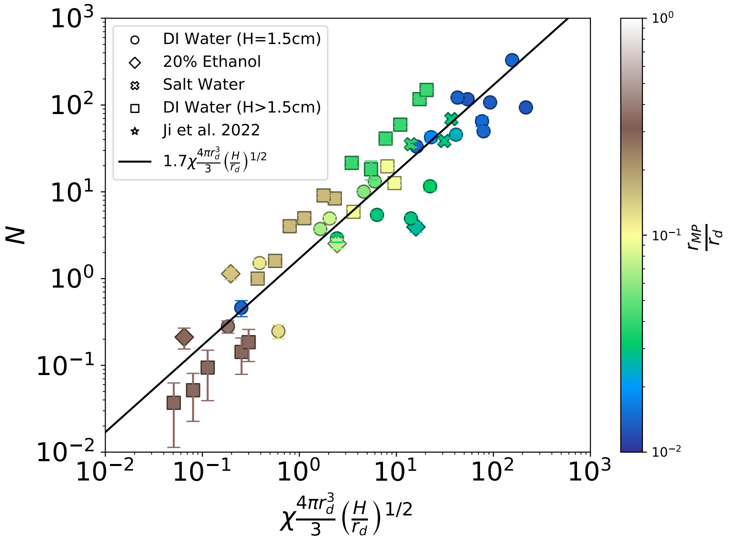

FIG. S7: The number of microplastic pieces per jet drop is presented. Since r_d is a unique function of R_b for a given l_μ [10], the efficiency factor can be represented as a function of H and r_d: $E(H, R_b) \equiv E(H, r_d) = E_1 \left[H/r_d(R_b/l_\mu)\right]^{1/2}$, with $E_1 \approx 1.7$ fitted to the data. This formulation is used to construct the global emission model which integrates over r_d.

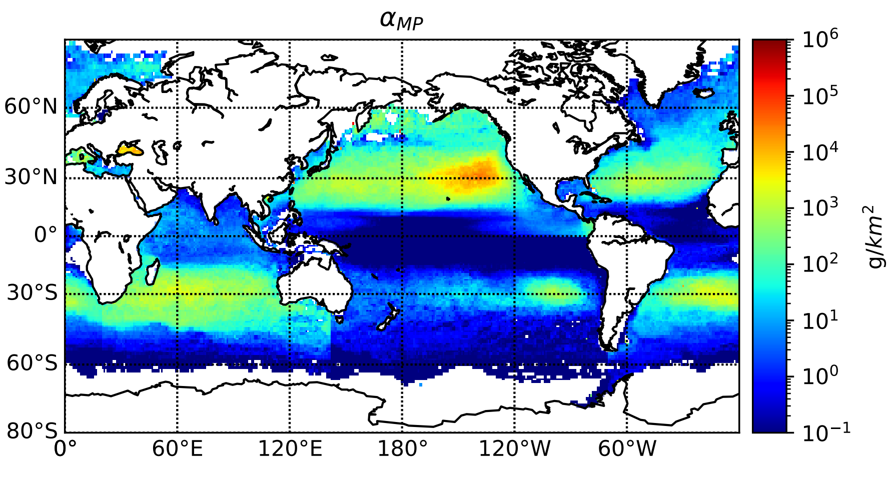

FIG. S8: Concentration of microplastic (330 μm < $2r_{MP}$ < 200 mm) by mass (g/km^2) as described by [22] employing the Maximenko model.

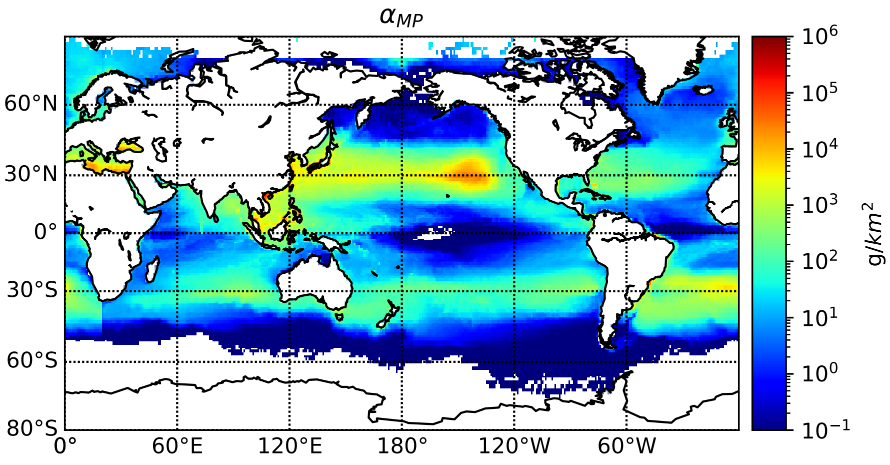

FIG. S9: Concentration of microplastic (330 µm < $2r_{MP}$ < 200 mm) by mass (g/km^2) as described by [22] employing the Lebreton model.

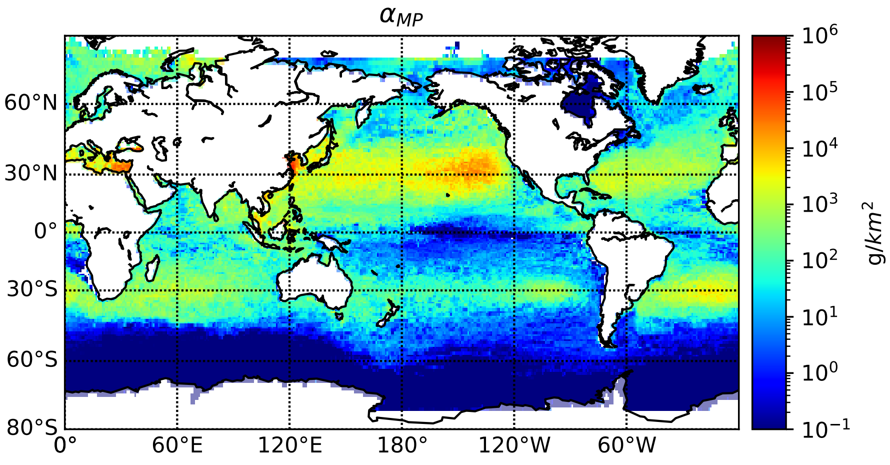

FIG. S10: Concentration of microplastic (330 µm < $2r_{MP}$ < 200 mm) by mass (g/km^2) as described by [22] employing the van Sebille model. The concentration of microplastic is an input to the global transport model.

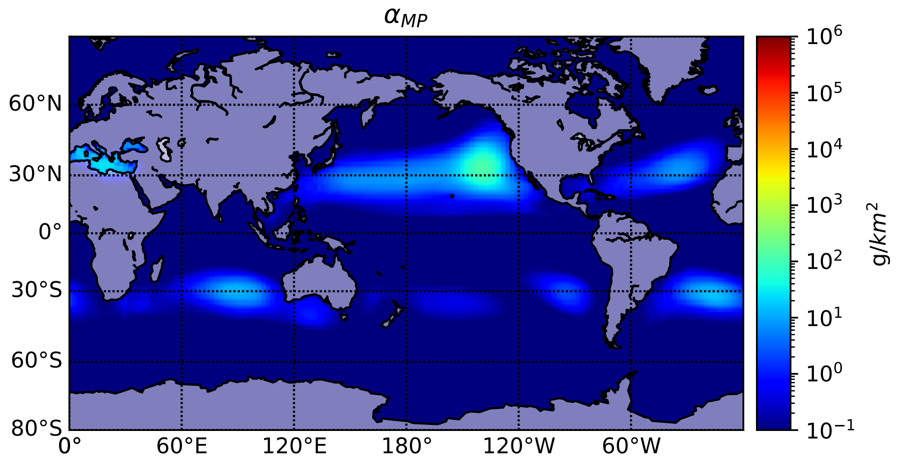

FIG. S11: Concentration of microplastic from [23] which has a particle size range of 0.3 µm < $2r_{MP}$ < 70 µm.

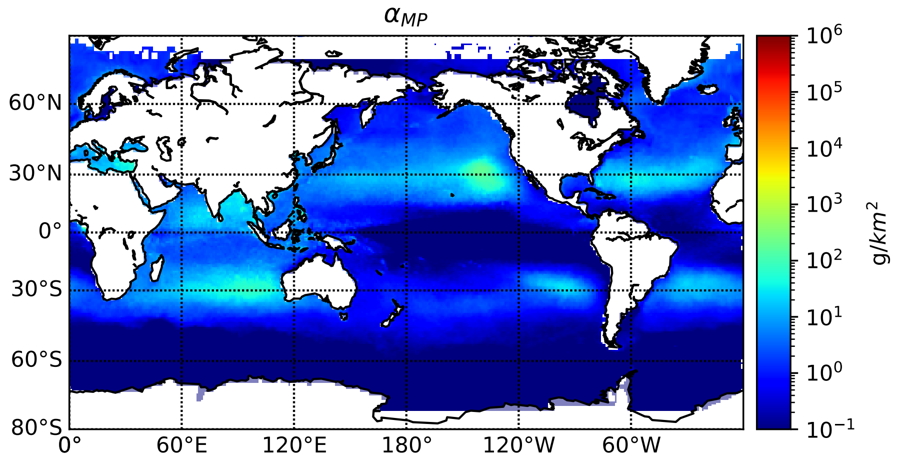

FIG. S12: Concentration of microplastic (100 µm < $2r_{MP}$ < 800 µ) in units of (g/km^2) located within 5m of the ocean surface in 2020 from [25]. The concentration of microplastic is an input to the global transport model.

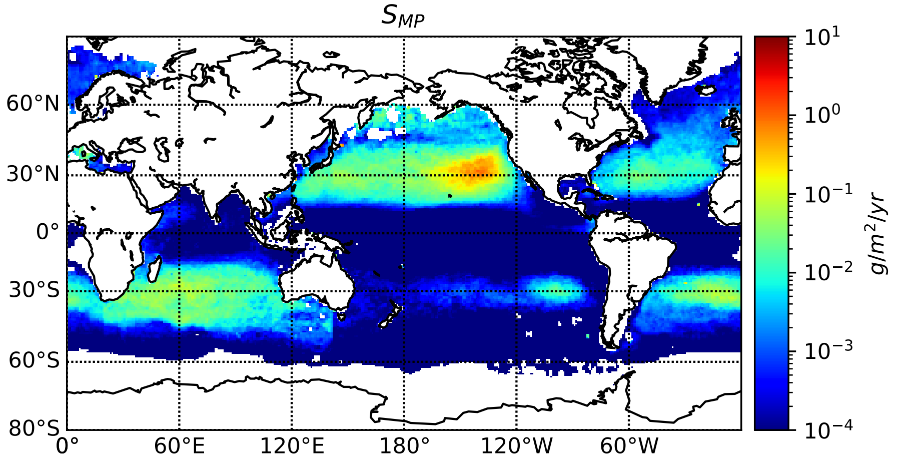

FIG. S13: Mass flux of microplastic from the ocean per unit area per unit time ($g/m^2/yr$) which is the output of the microplastic transport model in this work. This map uses the Maximenko transport model which has particle sizes of 330 μm $< 2r_{MP} <$ 200 mm for microplastic concentration data as input to the model.

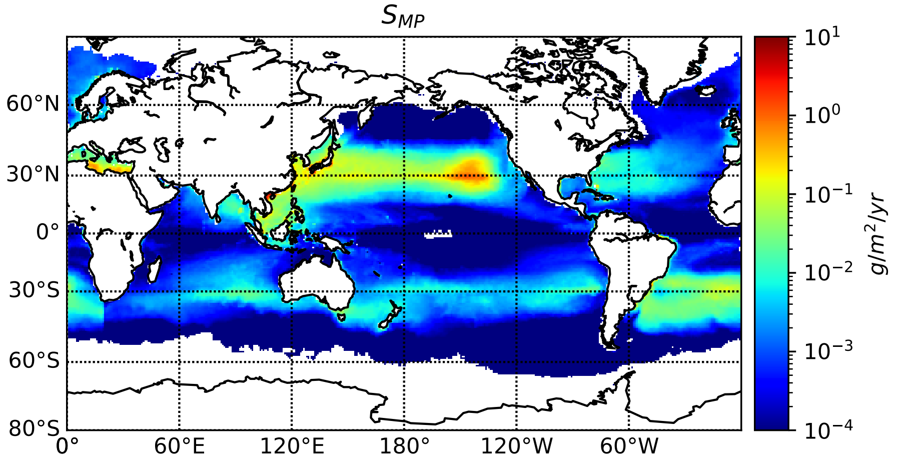

FIG. S14: Mass flux of microplastic from the ocean per unit area per unit time ($g/m^2/yr$) which is the output of the microplastic transport model in this work. This map uses the Lebreton transport model which has particle sizes of 330 μm $< 2r_{MP} <$ 200 mm for microplastic concentration data as input to the model.

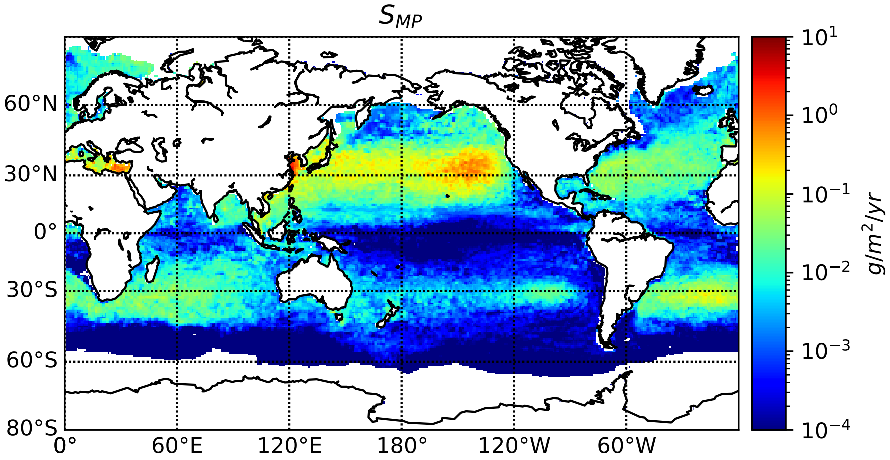

FIG. S15: Mass flux of microplastic from the ocean per unit area per unit time (g/m^2/yr) which is the output of the microplastic transport model in this work. This map uses the van Sebille transport model which has particles sizes of 330 µm $< 2r_{MP} <$ 200 mm for microplastic concentration data as input to the model.

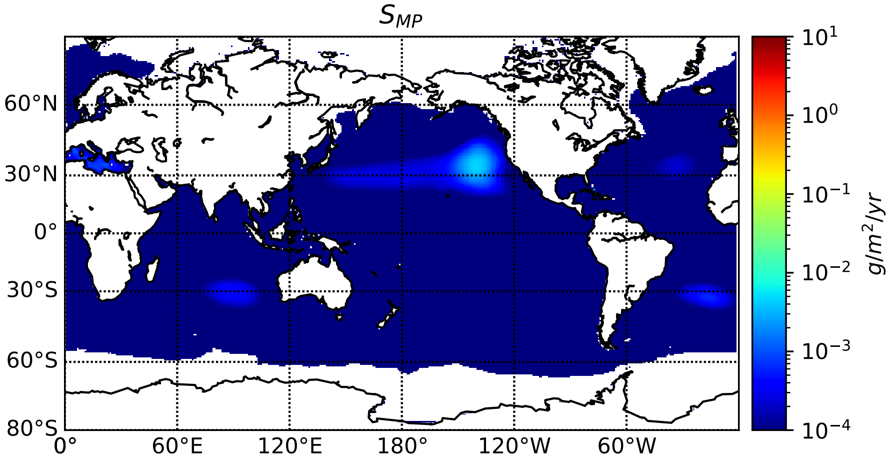

FIG. S16: Mass flux of microplastic using the concentration map from [23] which has a particle size range of 0.3 µm $< 2r_{MP} <$ 70 µm.

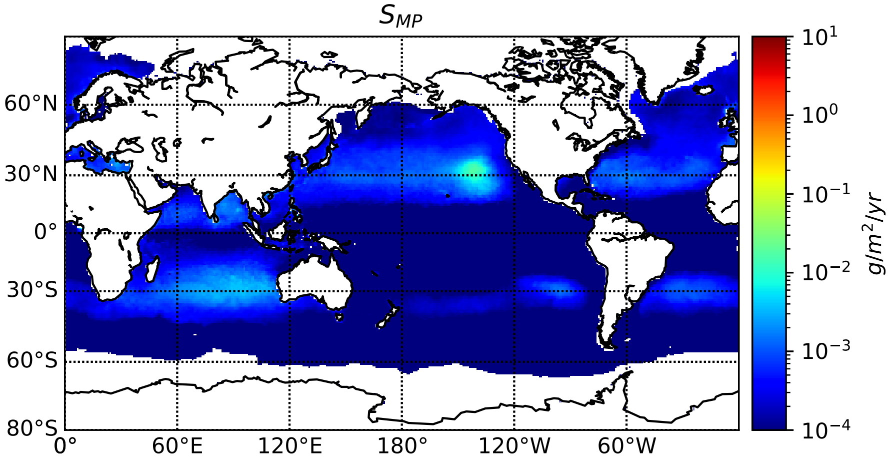

FIG. S17: Mass flux of microplastic using the concentration map from [25] which has a particle size range of 100 µm $< 2r_{MP} <$ 800 µm located in the upper 5m of the ocean in 2020.

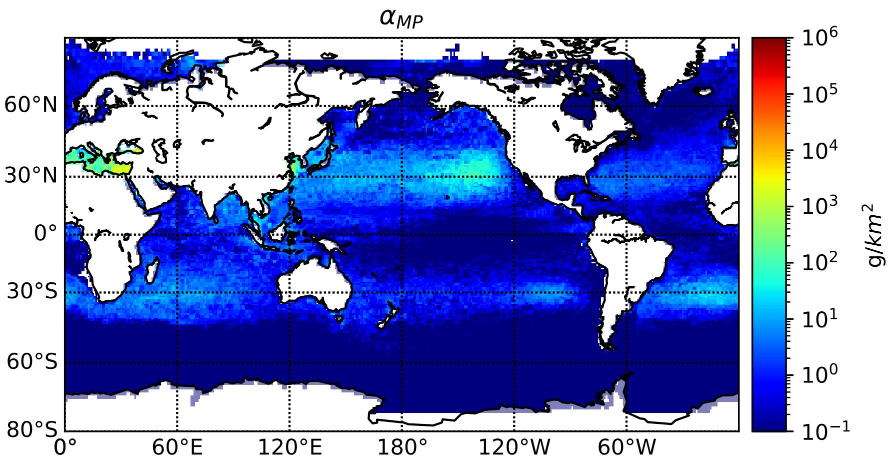

FIG. S18: Concentration of ejectable microplastic by assuming a particle size distribution of $P(r_{MP}) = Ae^{-r_{MP}/\langle r_{MP}\rangle}$ and integrating the dataset from [22] from 330 µm $< 2r_{MP} <$ 1 mm.

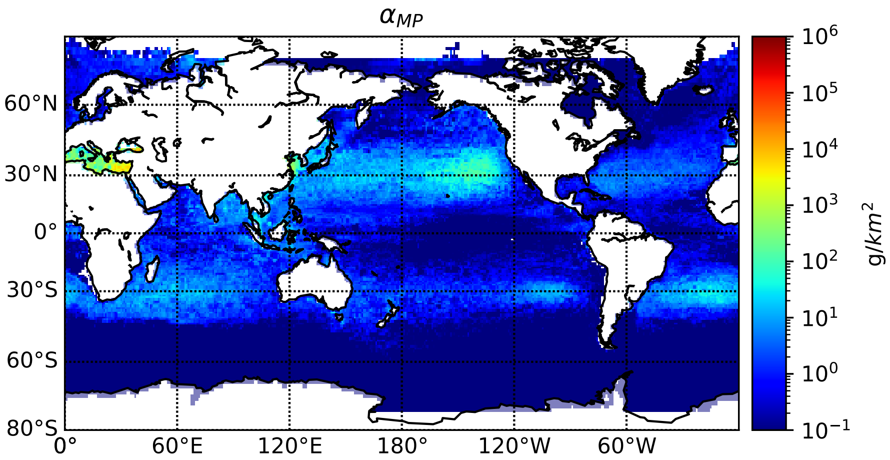

FIG. S19: Concentration of ejectable microplastic by assuming a particle size distribution of $P(r_{MP}) = \{B$ for $r_{MP} < \langle r_{MP}\rangle$ and C for $r_{MP} > \langle r_{MP}\rangle\}$ and integrating the dataset from [22] from 330 µm $< 2r_{MP} <$ 1 mm.

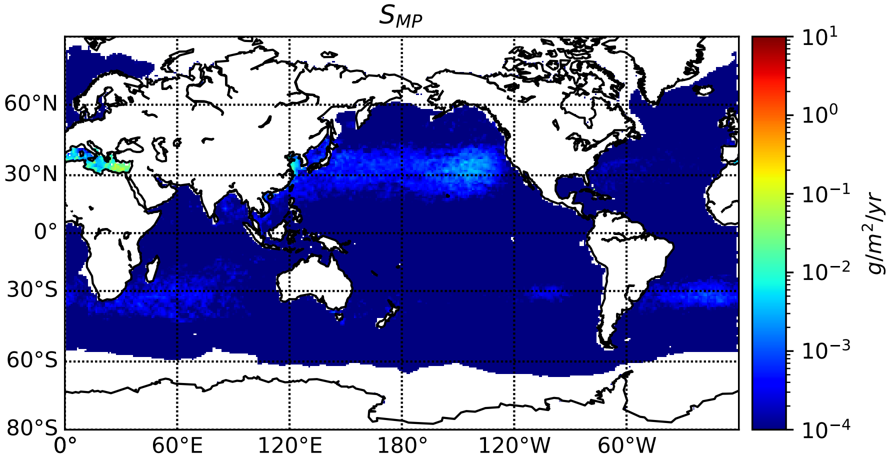

FIG. S20: Mass flux of microplastic from the ocean per unit area per unit time (g/m^2/yr) which is the output of the microplastic transport model in this work. This map uses concentration shown in Figure S18 which assumes $P(r_{MP}) = Ae^{-r_{MP}/\langle r_{MP}\rangle}$ and integrates the dataset of [22] from 330 µm $< 2r_{MP} <$ 1 mm.

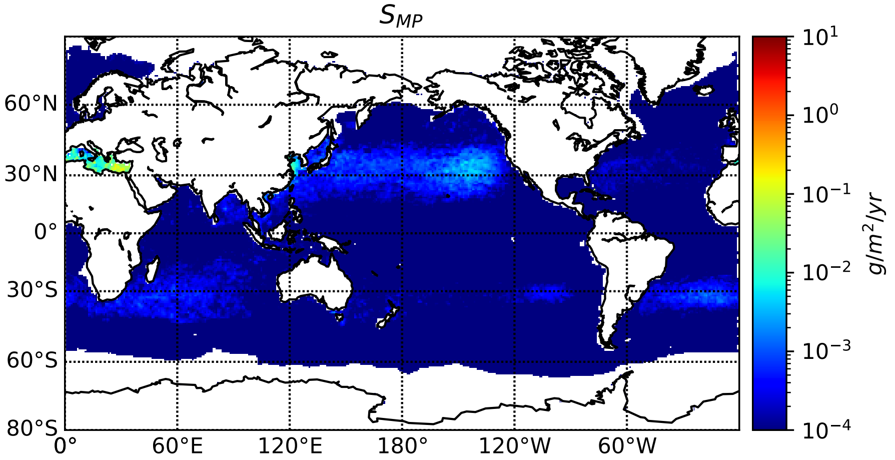

FIG. S21: Mass flux of microplastic from the ocean per unit area per unit time (g/m^2/yr) which is the output of the microplastic transport model in this work. This map uses concentration shown in Figure S19 which assumes $P(r_{MP}) = \{B$ for $r_{MP} < \langle r_{MP} \rangle$ and C for $r_{MP} > \langle r_{MP} \rangle\}$ and integrates the dataset of [22] from 330 µm $< 2r_{MP} <$ 1 mm.

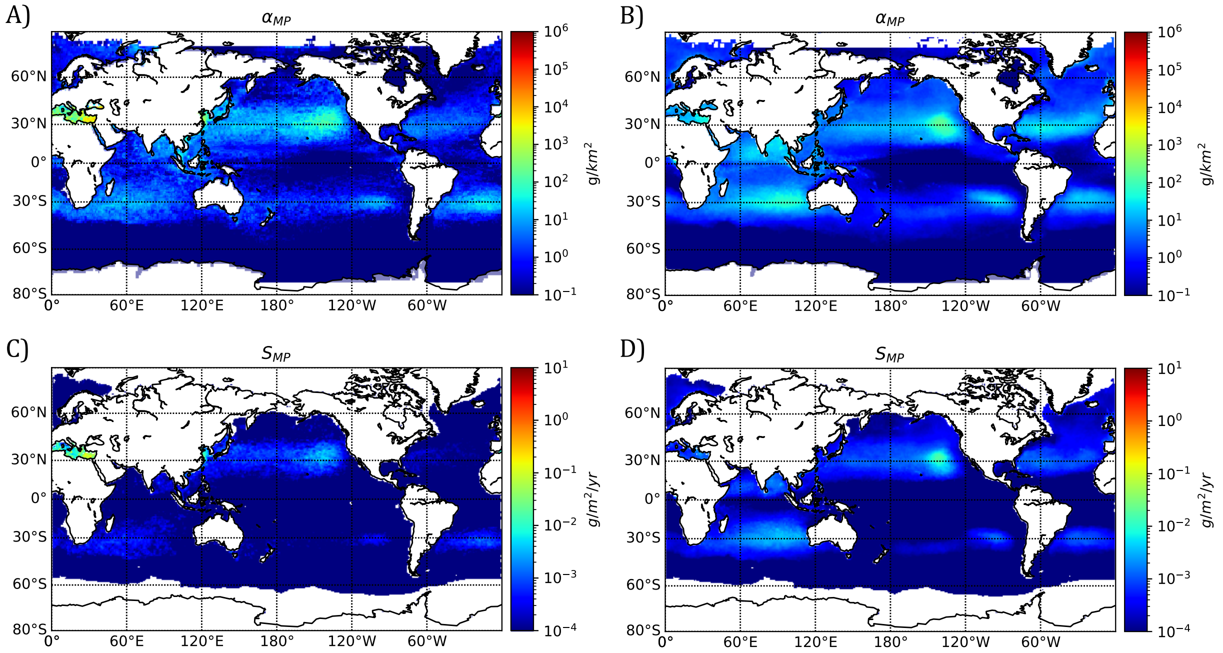

FIG. S22: Comparison of the concentration and mass flux of two different concentration maps. A) and B) show the same concentration maps as Figures S19 and S12 respectively. While the assumptions and models of each are different, the magnitude and distribution of each are very similar. C) and D) show the resulting mass flux rate with the concentration maps in of Figures S21 and S17 respectively. The total emission of each is 0.1 Mt/yr.

[1] D. Everett, *Basic Principles of Colloid Science*, ISSN (Royal Society of Chemistry, 2007).

[2] D. C. Blanchard, The Ejection of Drops from the Sea and Their Enrichment with Bacteria and Other Materials: A Review, Estuaries **12**, 127 (2006).

[3] Y. Toba, Drop Production by Bursting of Air Bubbles on the Sea Surface (II) Theoretical Study on the Shape of Floating Bubbles, Journal of the Oceanographical Society of Japan **15**, 121 (1959).

[4] H. Lhuissier and E. Villermaux, Bursting bubble aerosols, Journal of Fluid Mechanics **696**, 5 (2012).

[5] G. Taylor, The dynamics of thin sheets of fluid. III. Disintegration of fluid sheets, Proceedings of the Royal Society of London. Series A. Mathematical and Physical Sciences (1959).

[6] F. Culick, Comments on a ruptured soap film, Journal of Applied Physics **31**, 1128 (1960).

[7] A. M. Gañán-Calvo, Revision of Bubble Bursting: Universal Scaling Laws of Top Jet Drop Size and Speed, Physical Review Letters **119**, 1 (2017).

[8] A. Berny, S. Popinet, T. Séon, and L. Deike, Statistics of Jet Drop Production, Geophysical Research Letters **48**, 1 (2021).

[9] J. M. Gordillo and J. Rodríguez-Rodríguez, Capillary waves control the ejection of bubble bursting jets, Journal of Fluid Mechanics **867**, 556 (2019).

[10] F. J. Blanco-Rodríguez and J. M. Gordillo, On the sea spray aerosol originated from bubble bursting jets, Journal of Fluid Mechanics **886**, 1 (2020).

[11] A. Berny, L. Deike, T. Séon, and S. Popinet, Role of all jet drops in mass transfer from bursting bubbles, Physical Review Fluids **5**, 33605 (2020).

[12] D. E. Spiel, On the births of jet drops from bubbles bursting on water surfaces, Journal of Geophysical Research **100**, 4995 (1995).

[13] D. E. Spiel, The number and size of jet drops produced by air bubbles bursting on a fresh water surface, Journal of Geophysical Research **99**, 1028910296 (1994).

[14] L. Deike, B. G. Reichl, and F. Paulot, A mechanistic sea spray generation function based on the sea state and the physics of bubble bursting, AGU Advances **3**, e2022AV000750 (2022).

[15] F. Veron, Ocean spray, Annual Review of Fluid Mechanics **47**, 507 (2015).

[16] L. Deike, Mass Transfer at the Ocean–Atmosphere Interface: The Role of Wave Breaking,

Droplets, and Bubbles, Annual Review of Fluid Mechanics **54**, 191 (2022).

[17] E. Ghabache and T. Séon, Size of the top jet drop produced by bubble bursting, Physical Review Fluids **1**, 1 (2016), 1605.05876.

[18] C. F. Brasz, C. T. Bartlett, P. L. Walls, E. G. Flynn, Y. E. Yu, and J. C. Bird, Minimum size for the top jet drop from a bursting bubble, Physical Review Fluids **7**, 1 (2018).

[19] WW3DG, User manual and system documentation of wavewatch iii version 5.16. (tech. rep.). (2016).

[20] Japanese meteorological society reanalysis product (jra55-do).

[21] S. Kobayashi, Y. Ota, Y. Harada, A. Ebita, M. Moriya, H. Onoda, K. Onogi, H. Kamahori, C. Kobayashi, H. Endo, K. Miyaoka, and T. Kiyotoshi, The JRA-55 reanalysis: General specifications and basic characteristics, Journal of the Meteorological Society of Japan **93**, 5 (2015).

[22] E. van Sebille, C. Wilcox, L. Lebreton, N. Maximenko, B. D. Hardesty, J. A. Van Franeker, M. Eriksen, D. Siegel, F. Galgani, and K. L. Law, A global inventory of small floating plastic debris, Environmental Research Letters **10** (2015).

[23] J. Brahney, N. Mahowald, M. Prank, G. Cornwell, Z. Klimont, H. Matsui, and K. A. Prather, Constraining the atmospheric limb of the plastic cycle, Proceedings of the National Academy of Sciences of the United States of America **118**, 1 (2021).

[24] M. Eriksen, L. C. Lebreton, H. S. Carson, M. Thiel, C. J. Moore, J. C. Borerro, F. Galgani, P. G. Ryan, and J. Reisser, Plastic Pollution in the World's Oceans: More than 5 Trillion Plastic Pieces Weighing over 250,000 Tons Afloat at Sea, PLoS ONE **9**, 1 (2014).

[25] M. L. A. Kaandorp, D. Lobelle, C. Kehl, H. A. Dijkstra, and E. van Sebille, Global mass of buoyant marine plastics dominated by large long-lived debris, Nature Geoscience **16**, 689 (2023).
